# Targeted next-generation sequencing implementation in Eswatini identifies rifampicin and bedaquiline resistance undetected by routine diagnostic testing

**DOI:** 10.1038/s41467-026-73551-w

**Published:** 2026-06-11

**Authors:** Debrah Vambe, Alexander Kay, Mangaliso Ziyane, Mdigo Thunzini, Sein Sein Thi, Leonardo de Araujo, Viola Dreyer, Tanja Niemann, Busizwe Sibandze, Sijabu Masina, Andrea Maurizio Cabibbe, Jennifer Furin, Tafadzwa Mazuruse, Lindokuhle Dlamini, Thulani Jele, Bheki Mamba, Gugu Maphalala, Makhosazana Dlamini, Mbongeni Dube, Nomthandazo Lukhele, Daniela Maria Cirillo, Anna Mandalakas, Christoph Lange, Sphiwe Ngwenya, Christian Utpatel, Sindisiwe Dlamini, Stefan Niemann

**Affiliations:** 1 Baylor College of Medicine Children’s Foundation Eswatini, Mbabane, Eswatini; 2 Department of Paediatrics, Global TB Program, Baylor College of Medicine, Houston, TX USA; 3 National Tuberculosis Reference Laboratory, Eswatini Health Laboratory Services, Ministry of Health, Mbabane, Eswatini; 4 Department of Biomedical Sciences, Faculty of Health and Wellness Sciences, Cape Peninsula University of Technology, Bellville Campus, Bellville, South Africa; 5 National TB Control Program, Manzini, Eswatini; 6 Research Centre Borstel Leibniz Lung Center, Molecular and Experimental Mycobacteriology, Borstel, Germany; 7 German Center for Infection Research (DZIF), Partner Site Hamburg-Lübeck-Borstel-Riems, Borstel, Germany; 8 IRCCS San Raffaele Scientific Institute, Emerging Bacterial Pathogens Unit, Milan, Italy; 9 Harvard Medical School, Department of Global Health and Social Medicine, Boston, MA USA; 10 Good Shepherd Hospital, Siteki, Eswatini; 11 Nhlangano Health Centre, TB Reference Hospital, Ministry of Health, Mbabane, Eswatini; 12 World Health Organization Country Office, Mbabane, Eswatini; 13 Respiratory Medicine & International Health, University of Lübeck, Lübeck, Germany; 14 Clinical Infectious Diseases, Research Center Borstel, Leibniz Lung Center, Borstel, Germany; 15 Clinical Tuberculosis Unit (ClinTB), German Center for Infection Research (DZIF), Hamburg-Lübeck-Borstel-Riems, Germany; 16 Phylogenomics, Phylodynamics and Host-Pathogen Interactions team, EPHE, PSL University, Paris, France; 17 Institut de Systématique, Evolution, Biodiversité (ISYEB), MNHN, CNRS, SU, UA, EPHE, Paris, France

**Keywords:** Tuberculosis, Infectious-disease diagnostics, Antimicrobial resistance

## Abstract

In Eswatini, multidrug-resistant (MDR) *Mycobacterium tuberculosis* (Mtb) strains harbouring the rifampicin-resistance (RR) *rpoB* I491F mutation are missed by routine diagnostics, including GeneXpert MTB/RIF Ultra (Xpert Ultra), line probe assays (LPA), and mycobacterium growth indicator tube (MGIT) phenotypic drug susceptibility testing (pDST). To address this diagnostic gap, Eswatini introduced targeted next-generation sequencing (tNGS) in 2019. We analysed 234 patient samples enrolled from June 2021 to December 2024 with isoniazid and/or rifampicin resistance detected by routine diagnostics, or suspected treatment failure, and obtained detailed clinical and outcome data from 59 patients. tNGS detected RR in 159 strains, of which 101 (64%) carried the *rpoB* I491F mutation. Bedaquiline (BDQ) resistance, conferred by *Rv0678* mutations, was identified in 87 strains, rendering 55% (87/159) of RR and 85% (86/101) of *rpoB* I491F strains genotypically BDQ-resistant. Routine tests substantially under-classified resistance, particularly in strains reported as isoniazid-resistant and rifampicin-susceptible. tNGS-informed treatment changes occurred in 53% (31/59) of patients, with 88% (52/59) treatment success. tNGS is therefore an essential tool to detect *rpoB* I491F “diagnostic escape” strains with additional BDQ resistance, and underscores the urgent need to reconsider current BPaLM regimens and global drug-resistance classifications.

## Abstract (web summary)

Routine diagnostic tests in Eswatini miss *Mycobacterium tuberculosis* resistance to key drugs. Here the authors show that targeted next-generation sequencing identifies these elusive strains, guiding personalized treatment and achieving an 88% treatment success rate.

## Introduction

An estimated 390,000 (95% uncertainty interval [UI]: 360,000–430,000) people developed rifampicin-resistant (RR) or multidrug-resistant (at least resistant to rifampicin and isoniazid) (MDR) tuberculosis (TB) in 2024, but only 42% were identified and initiated treatment^1^. While global treatment success rates have improved, only 71% of people with MDR/RR have favourable treatment outcomes^1^, and most experience delays in accessing appropriate treatment^2^.

Eswatini is one of the 30 high TB/HIV burden countries, with a tuberculosis incidence of 319/100,000, and MDR/RR TB of 3.2% among new patients and 25.3% among previously treated patients^1^. To increase access to drug-resistant-tuberculosis care and promote person-centred services, Eswatini decentralised drug-resistant-tuberculosis services, adopted new drugs and shorter regimens, and offers a comprehensive patient support package (monthly food packages, transport allowance, stipend for treatment supporters, treatment literacy, and psychological counselling). These changes contributed to a treatment success rate of 86% in patients with drug-resistant tuberculosis. Despite these strategies and good treatment success rates for those identified as being affected by drug-resistant tuberculosis, the country still does not detect 67% of people with MDR/RR tuberculosis^3^.

This is largely attributable to an epidemic drug-resistant *Mycobacterium tuberculosis* (Mtb) strain with a specific rifampicin resistance mutation in *rpoB* (namely I491F). This mutation is classified as a low-level mutation in the WHO catalogue^4^. Resistance related to this mutation is difficult to detect using conventional phenotypic drug susceptibility testing (pDST) with mycobacterium growth indicator tube (MGIT) (BD, Sparks, MD) pDST with WHO-recommended critical concentrations. Mtb strains with *rpoB* I491F mutation are also not detected by currently WHO endorsed molecular rifampicin-resistance detection assays such as GeneXpert® MTB/RIF Ultra (Cepheid, Sunnyvale, CA) and the GenoType MTBDRplus (Bruker-Hain Lifescience, Nehren, Germany) line probe assay (LPA)^5–7^. This mutation was detected in 30% of Mtb strains obtained from MDR/RR tuberculosis patients during a national drug resistance survey in 2009^6^, and increased to 58% of MDR/RR tuberculosis patients in the 2018 national survey^8^. Of substantial public health significance is the fact that a notable proportion of this strain also carries mutations associated with resistance to bedaquiline and clofazimine^7^.

While rapid and comprehensive drug resistance prediction, including rifampicin resistance due to the *rpoB* I491F mutation and bedaquiline/clofazimine resistance due to Rv0678 mutations, has become possible with the introduction of targeted next-generation sequencing (tNGS) methods^7,9–11^, their implementation for clinical use has not occurred in many high-burden countries^12^. In response to the emerging threat in Eswatini, the Eswatini National Tuberculosis Control Program (NTCP) and Eswatini Health Laboratory Services (EHLS) initiated the development of a tNGS program in 2019.

Here, we describe the processes followed to develop the tNGS program in the country, early experiences with programmatic implementation, and the added diagnostic value of tNGS in DR-TB compared with routine diagnostic methods.

## Results

### Program development and clinical integration

**Fig. 1:**
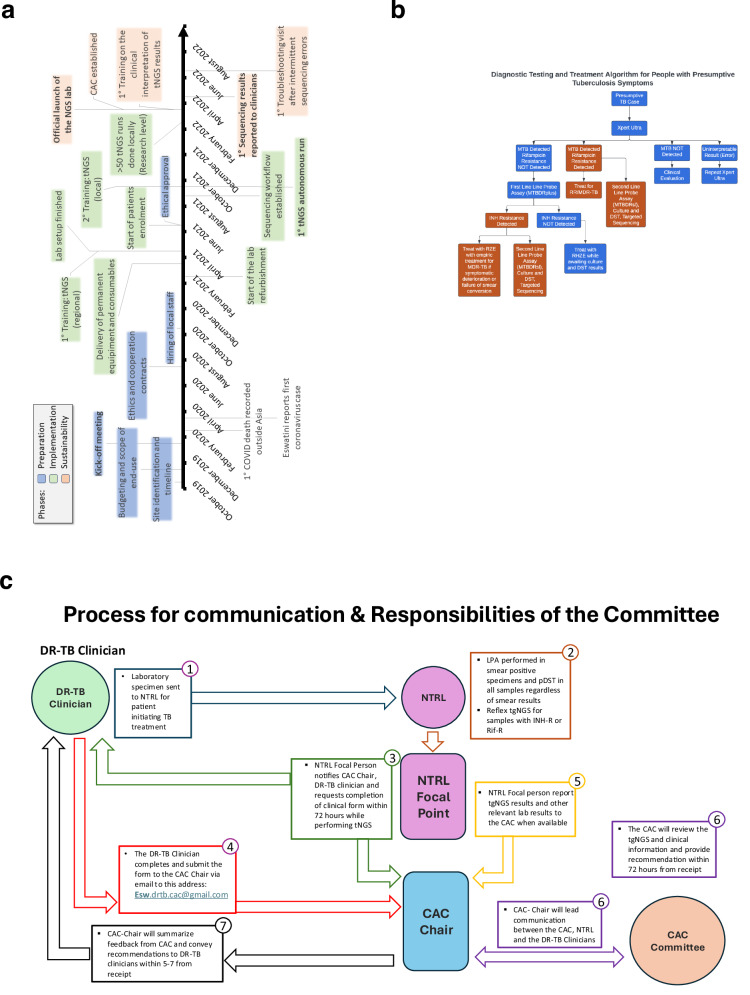
a–c. Programmatic implementation of targeted next-generation sequencing (tNGS) for drug-resistant tuberculosis at the Eswatini National Tuberculosis Reference Laboratory: Timeline, Diagnostic Algorithm, and Clinical Advisory Committee workflow. **a** Timeline depicting the phased implementation of targeted next-generation sequencing (tNGS) at the National TB Reference Laboratory in Mbabane, Eswatini. Our approach to tNGS adoption followed a structured framework encompassing the preparation, implementation, and sustainability phases. The official initiation of the implementation initiative commenced on January 01, 2020. The first funding phase concluded on 31 December 2022. Notably, regional training sessions were conducted at the University of Namibia, necessitated by travel restrictions imposed during the SARS-CoV-2 pandemic. **b** The Eswatini Diagnostic Testing and Treatment Algorithm for people with presumptive tuberculosis symptoms uses a stepwise approach to guide tuberculosis diagnostic testing and make treatment decisions. The orange boxes reflect the algorithmic responses to resistance detection. The blue boxes reflect the algorithmic responses to susceptible or indeterminate results. **c** Clinical Advisory Committee (CAC) flow diagram outlining the roles and responsibilities of the clinicians, laboratory team, Clinical Advisory Committee members, and tuberculosis program (Clinical Advisory Committee chair) for the tNGS program.

Local implementation of tNGS was initiated in 2019 and launched in January 2020 through a collaborative project funded by the Global Health Protection Program (GHPP) of the German Ministry of Health. The launch of the tNGS program coincided with the COVID-19 pandemic, which led to delays in the planned renovation of the future NGS laboratory infrastructure and supply shipments (Fig. 1A). However, with support from the German Ministry of Health and guidance from the Molecular and Experimental Mycobacteriology (MolMyc) group and WHO Supranational Tuberculosis Reference Laboratory at the Research Center Borstel, Germany, Eswatini’s NTRL was redesigned to support tNGS. A sequencing team was hired, Illumina iSeq100 sequencers were procured, and reagents were obtained. The sequencing team was trained in Eswatini and at regional partner laboratories by a team from MolMyc, WHO Supranational Tuberculosis Reference Laboratories in Borstel and Milan. The redesigned NTRL NGS lab opened in April 2021, just under two years after the project launch (Fig. 1A). The NTCP collaborated closely with the WHO office in Eswatini and the WHO Global Tuberculosis Program on the integration of sequencing into clinical algorithms in advance of the WHO guidance on the programmatic use of tNGS. Eswatini aligned result interpretation with the WHO catalogue of mutations launched in 2021 to define tNGS resistance classifications, which was further updated in 2023^4^ and supplemented with the Research Center Borstel catalogue for rare mutations that were not fully addressed in the WHO catalogue.

While establishing the laboratory infrastructure for tNGS, the NTCP revised the diagnostic and treatment guidelines to include tNGS in the country’s diagnostic algorithm. It was clear that at the initial implementation stage, sequencing capacity would not support universal tNGS of all drug-resistant isolates; however, the 2018 national drug resistance survey established that Mtb strains with the *rpoB* I491F mutation leading to rifampicin-resistance also had isoniazid resistance^8^. Therefore, tNGS was included in the diagnostic algorithm for Mtb strains identified as having rifampicin or isoniazid resistance detected by LPA, pDST, or Xpert Ultra testing (Fig. 1B). Clinicians could also request tNGS for patients who are not clinically improving during therapy or at the time of diagnosis if they are potentially exposed to drug-resistant tuberculosis.

Orientation and ongoing engagement of clinicians on the use of tNGS results were promoted in bi-weekly national drug-resistant TB clinical meetings established by the NTCP. A CAC was established on March 20th, 2022, with terms of reference and standard operating procedures (SOPs) (Supplemental Data), followed by the first training of clinicians on interpretation of tuberculosis tNGS results with the support of the MolMyc group. The CAC comprised Eswatini-based drug-resistant tuberculosis clinicians and external drug-resistant tuberculosis experts with clinical, laboratory, and public health expertise. The CAC provided guidance to clinicians and the NTCP on the interpretation and management of patients with additional sequencing results. Job aids and SOPs were developed to support implementation, coordinate data transfer, and define expectations for clinicians, laboratory staff, NTCP officials, and CAC members (Fig. 1C). The first sequencing results were reported to the clinicians on 1 April 2022.

### Diagnostic reclassification by targeted next-generation sequencing

Here, we present data on drug resistance classifications based on tNGS compared to routine diagnostics for 234 Mtb strains and the impact of incorporating tNGS into diagnostic and clinical treatment algorithms in Eswatini among 59 patients with complete clinical data.

This cohort represents 53% (234/442) of patients treated for drug-resistant TB in Eswatini from June 2021 to December 2024 (Table 1). The cohort had 65% males, which was comparable to the national data between June 2021 and December 2024 for drug-resistant TB (63%), and had a median age of 37 years, which was also similar to the average median age of clients treated for drug-resistant TB in Eswatini (35–39 years) (Table 1). HIV status was collected for 145 patients in the cohort and was positive in 64.8% of patients, again in line with the national prevalence (61%) of HIV in people with DR-TB (Table 1). The regional distribution of DR-TB in this patient cohort mirrored the national data.

**Table 1:** Demographics of sequencing and June 2021–December 2024 Eswatini DR-TB cohort

	June 2021–December 2024 Sequencing Cohort (n = 234)	June 2021–December 2024 DR-TB Cohort (n = 442)
Age, Median (IQR)	37.0 years (28–48)	35–39 years (median group) †
Male sex (%) (n = 233)	153 (65.4%)	279 (63.1%)
Region		
Hhohho	68 (29.1%)	Hhohho 100 (22.6%)
Lubombo	38 (16.2%)	Lubombo 76 (17.2%)
Manzini	91 (38.9%)	Manzini 195 (44.1%)
Shiselweni	37 (15.8%)	Shiselweni 71 (16.1%)
HIV Status (n = 145)		
Non-reactive	51 (35.2%)	Non-reactive 172 (38.9%)
Reactive	94 (64.8%)	Reactive 270 (61.1%)

Abbreviations: IQR; inter-quartile range, TB; tuberculosis.

† Exact IQR not available; age provided as median age group.

**Fig. 2:**
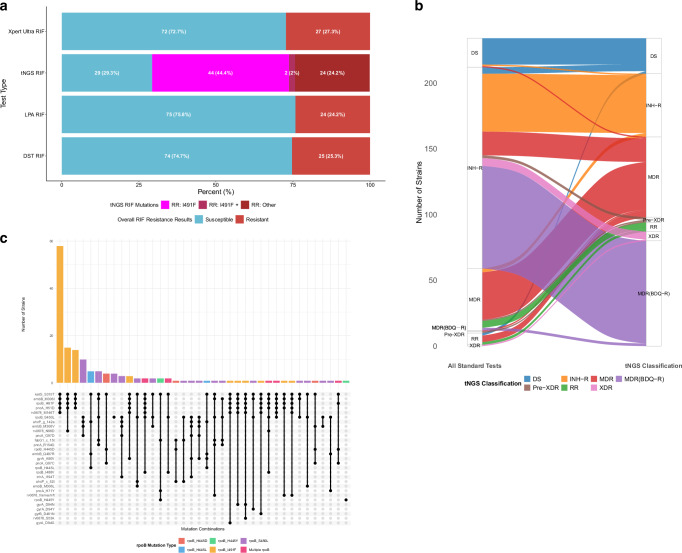
a–c. tNGS reveals missed rifampicin resistance, reclassifies drug-resistance categories, and maps dominant mutation profiles in the Eswatini cohort. **a** Results of rifampicin resistance testing among 99 participants in whom Xpert Ultra, tNGS, LPA and DST, were performed and generated valid results, demonstrating the clear discrepancy between tNGS for detection of rifampicin resistance vs. all other methods due to the presence of the *rpoB* I491F mutation. Abbreviations: LPA; line probe assay, tNGS; targeted next generation sequencing, DST; drug susceptibility testing, RIF; rifampicin. **b** The left pillar of the alluvial plot demonstrates the baseline classification of WHO tuberculosis drug resistance classifications (drug-susceptible TB as defined by the absence of resistance to isoniazid and rifampicin, isoniazid-resistant TB, rifampicin-resistant TB, multidrug-resistant TB with and without bedaquiline resistance, pre-extensively drug-resistant TB, and extensively drug-resistant TB), using results for isoniazid, rifampicin, fluoroquinolones, bedaquiline, and linezolid based on the results of Xpert Ultra, LPA, XDR, or MGIT DST. We included an additional classification to address the presence of bedaquiline resistance without fluoroquinolone resistance. Resistance classifications were made based on a worst-case approach in the event of conflicting results. The 234 participants included had at least one valid result for Xpert Ultra, LPA, Xpert XDR, or MGIT DST but did not have each test type performed. The right pillar demonstrates the WHO drug resistance classification following the incorporation of tNGS results; all 234 participants had valid tNGS results. Abbreviations: DS; drug susceptible, INH-R; resistance to isoniazid only, MDR; multidrug resistant, RR; rifampicin resistant without evidence of other drug resistance, MDR (BDQ-R); MDR and resistance to bedaquiline, Pre-XDR; MDR and resistance to a fluoroquinolone, XDR; Pre-XDR and resistance to bedaquiline or linezolid. **c** This upset plot displays strains with sets of mutations according to the frequency of occurrence. The plot includes strains with *rpoB* mutations and mutations to INH, RIF, EMB, and PZA that were identified in at least three strains (n = 154 strains). The plot includes all mutations in FQ, BDQ, or LNZ that were identified, without restriction by the frequency of occurrence. The vertical bars show the total number identified for each pattern of mutations detected within individual patients and are colour-coded by the *rpoB* mutation identified.

Within this group of 234 individuals with TB, tNGS detected RR in 68% (159/234) of individuals, compared to 32.3% (43/133) for Xpert Ultra, 19.5% (37/190) for LPA, and 19.6% (42/214) for MGIT pDST (Supplemental Table 1). For 99 strains with results available for all tests, tNGS detected RR in 71% (70/99) of cases, compared to 27% (27/99) for Xpert Ultra, 24% (24/99) for LPA, and 25% (25/99) for MGIT pDST (Fig. 2A). Of the 159 RR TB patients, 101 (64%) had the *rpoB* I491F mutation (96 solo and 5 with other known RR mutations), aligning with the low rates of resistance detection seen with Xpert Ultra, LPA, and MGIT pDST (Supplemental Tables 1 and 2).

Figure 2B shows the composite drug-resistant WHO TB classifications (drug-susceptible TB (defined as the absence of resistance to isoniazid and rifampicin), isoniazid-resistant TB, rifampicin-resistant TB, multidrug-resistant TB with and without bedaquiline resistance, pre-extensively drug-resistant TB, and extensively drug-resistant TB, using results for isoniazid, rifampicin, fluoroquinolones, bedaquiline, and linezolid) defined by the ‘worst case’ result from Xpert Ultra, Xpert XDR, LPA, or MGIT pDST, followed by reclassification with tNGS results. The classification proportions and concordance, as measured by this composite metric, and each individual routine diagnostic test and tNGS are displayed in Table 2. Among strains classified by routine diagnostics as drug susceptible, 9% (2/22) were reclassified, one as isoniazid mono-resistance and one as MDR. There were 153 isolates initially defined as isoniazid mono-resistant by routine diagnostics, 3% (5/153) of which were reclassified as DS, 12% (18/153) as MDR, 51% (78/153) as MDR with bedaquiline resistance, 1% (2/153) as pre-XDR, 4% (6/153) as XDR, and 29% (44/153) were not reclassified (Supplemental Table 3). Overall, 87 isolates were classified as BDQ resistant, of which 69 had a Rv0678 M146T and 16 had Rv0678 N98D mutation, all of which were found in MDR strains with *rpoB* I491F mutation (81 with I491F mutation only, four with an I491F|S450L double mutation) (Supplemental Table 2).

**Table 2:** WHO resistance classifications by test

Resistance Classification	Xpert Ultra N = 133	Xpert XDR N = 10	LPA^2^ N = 192	MGIT^3^ N = 205	Composite (routine tests) N = 234	tNGS N = 234
DS^1^	90 (67.6%)	0	22 (11.5)	24 (11.7)	22 (9.4)	27 (11.5)
INH-R	NA	10 (100%)	133 (69.3)	139 (67.8)	153 (65.4)	48 (20.5)
RR	43 (32.3%)	NA	15 (7.8)	3 (1.5)	9 (3.8)	7 (3.0)
MDR	NA	NA	21 (10.9)	37 (18.0%)	47 (20.1)	61 (26.1)
MDR (BDQ)	NA	NA	NA	0	1 (0.4)	80 (34.2)
Pre-XDR	NA	0	1 (0.5%)	1 (1.3)	1 (0.4)	4 (1.7)
XDR	NA	NA	0	1 (1.3)	1 (0.4)	7 (3.0)
Agreement with tNGS^4^	46 (34.6%)		82 (42.7%)	89 (43.4%)	104/234 (44.4%)	
Agreement by recommended treatment regimens^5^			91 (47.4%)	94 (45.9%)	114 (48.7%)	
Agreement with tNGS (including only those with 1st and 2nd line test results)			N = 144 58 (40.3%)	N = 76 40 (52.6)		

^1^DS TB is defined as the absence of isoniazid and rifampicin resistance according to WHO classifications.

^2^Includes 144 patients with LPA fluoroquinolone results.

^3^Includes 76 patients with MGIT BDQ and FQ results.

^4^Agreement is for RR or MDR results.

^5^Global treatment recommendations are the same for RR and MDR TB.

Of 56 isolates identified as MDR/RR by routine diagnostics, tNGS identified additional resistance to bedaquiline in 4% (2/56) and fluoroquinolones in 2% (1/56); 5% (3/56) were reclassified as isoniazid mono-resistant, 4% (2/56) as DS, and 86% (48/56) were not reclassified. One strain was identified as MDR with bedaquiline resistance by routine testing, which was reclassified as MDR by tNGS. One strain was classified as pre-XDR and one as XDR, both of which were confirmed by tNGS (Supplemental Table 3). When limited to 76 patients with complete DST results for rifampicin, isoniazid, fluoroquinolones, and bedaquiline, high rates of resistance misclassification by routine diagnostics were still observed (Supplemental Fig. 1).

Bedaquiline susceptibility results obtained using MGIT pDST and tNGS were discordant when both tests were performed with valid results (N = 84). Resistance to bedaquiline was identified in five strains by pDST, four of which were also resistant by tNGS. However, 22 strains with bedaquiline resistance identified by tNGS were tested as susceptible by MGIT pDST using the single critical concentration recommended by the WHO version 2, and 57 strains were susceptible by both measures (Supplemental Table 4). Of the 22 strains with mutations associated with BDQ resistance identified by tNGS, all were in the Rv0678 gene, and 20 were classified by the WHO as resistant (Rv0678 M146T). The remaining two mutations (N98D), although not classified as resistant by the WHO catalogue v2, were associated with elevated MICs to BDQ in our previous work^5,13^.

Figure 2C demonstrates the most common patterns of resistance identified. Mutations in Rv0678 were detected in 37% (87/234) of all isolates, 55% (87/159) of rifampicin-resistant isolates, and 85% (86/101) of Mtb strains with the *rpoB* I491F mutation (Supplemental Table 2). Overall, mutations associated with fluoroquinolone resistance were detected in 5% (11/234) of the strains investigated. Notably, seven strains with bedaquiline and clofazimine resistance due to a mutation in Rv0678 had additional mutations associated with resistance to fluoroquinolones and were classified as XDR. We compared standardised treatment regimens derived from routine diagnostics with those derived from tNGS. Only 35% of regimens (46/133) based on Xpert® MTB/RIF, 47% (91/192) on LPA, and 46% (94/205) on MGIT pDST derived from WHO guideline-based treatment algorithms would have been administered correctly, despite the presence or absence of drug resistance as detected by tNGS results (Table 2, Supplemental Table 5A–F).

### Clinical use of sequencing results and treatment implications

Complete clinical information was obtained from 59 patients with tNGS results (Table 3). The CAC received sequencing results for all 59 patients and a patient report form from the DR-TB clinician for 81% (48/59) of the patients. Some clinicians did not submit the forms, opting instead to continue with the prescribed regimens they considered effective. The CAC reviewed the patient data and provided recommendations for 96% (46/48) of the patients for whom a complete report was shared. A change in treatment was recommended in 26 (54%, one died prior to this recommendation), 20 (42%) were advised to continue the same regimen, and no recommendation was provided for 2 (4%). Based on the tNGS results, 31/59 patients (53%) had their regimens changed. Of these, 25 changes were made following CAC recommendations, while clinicians independently modified treatment in six patients, suggesting increased capacity and confidence among the treating clinicians.

**Table 3:** Treatment regimens and outcomes of patients based on tNGS results, treating clinicians, and Clinical Advisory Committee recommendations

Characteristics	Category/regimen	Overall (N = 59)
Age (years)	Median [Min, Max]	38.0 [14.0, 76.0]
Sex	Female	21 (36%)
	Male	38 (64%)
HIV Status	Negative	18 (31%)
	Positive	40 (68%)
	Unknown	1 (2%)
Previously Treated for TB	No	42 (71%)
	Yes	17 (29%)
Prior TB Drug Susceptibility (n = 17)	DR-TB	2 (12%)
	DS-TB	15 (88%)
Committee Submission Complete	No	11 (19%)
	Yes	48 (81%)
Committee Recommendation (n = 48)	Change	26 (54%)
	No Change	20 (42%)
	Missing	2 (4%)
Regimen Changed Due to tNGS	Changed	31 (53%)
	No Change	28 (47%)
Who prompted the change	CAC	25 (42%)
	Clinician	6 (10%)
	No Change	28 (47%)
Original TB Regimen	BDQ, DLM, CFZ, TZD	1 (2%)
	BDQ, LFX, LZD, CFZ	1 (2%)
	BDQ, LFX, LZD, DLM, CFZ	8 (14%)
	BDQ, Pa, LZD, MFX	4 (7%)
	RHZE	45 (76%)
Empiric Regimens pre-tNGS	BDQ, DLM, CFZ, TZD	1 (2%)
	BDQ, LFX, DLM, CFZ, TZD	3 (5%)
	BDQ, LFX, LZD, CFZ	1 (2%)
	BDQ, LFX, LZD, CFZ, PTA	1 (2%)
	BDQ, LFX, LZD, CFZ, TZD	7 (12%)
	BDQ, LFX, LZD, DLM, CFZ	18 (31%)
	BDQ, LFX, LZD, DLM, TZD	1 (2%)
	BDQ, Pa, LZD, MFX	16 (27%)
	RHZE	11 (19%)
tNGS Informed Regimen	BDQ, LFX, DLM, CFZ, TZD	2 (3%)
	BDQ, LFX, LZD, CFZ, PTA	1 (2%)
	BDQ, LFX, LZD, CFZ, TZD	4 (7%)
	BDQ, LFX, LZD, DLM, CFZ	19 (32%)
	BDQ, LZD, DLM, CFZ, TZD	1 (2%)
	BDQ, Pa, LZD, MFX	7 (12%)
	DLM, PTA, IMP, TZD	1 (2%)
	LFX, LZD, DLM, IMP, TZD	2 (3%)
	LFX, LZD, DLM, PAS, IMP, AMK, TZD	1 (2%)
	LFX, LZD, DLM, PAS, IMP, TZD	1 (2%)
	LFX, LZD, DLM, PTA, IMP, TZD	2 (3%)
	LFX, LZD, DLM, PTA, TZD	4 (7%)
	LFX, LZD, DLM, PZA	1 (2%)
	LFX, LZD, DLM, TZD	1 (2%)
	LFX, LZD, DLM, TZD, PZA	6 (10%)
	LFX, RHZE	2 (3%)
	LZD, DLM, PTA, PAS, IMP, TZD	1 (2%)
	RHZE	3 (5%)
Treatment Outcome	Cured	18 (31%)
	Died	5 (8%)
	Lost to Follow-up	2 (3%)
	Treatment Completed	34 (58%)

Abbreviations: R: rifampicin; H: isoniazid, Z: pyrazinamide; E: ethambutol; BDQ: bedaquiline; LFX: levofloxacin; LZD: linezolid; CFZ: clofazimine; ETO: ethionamide; DLM: delamanid; TRD: terizidone; Pa: pretomanid; PTO: prothionamide; IMP-CLV: imipenem-cilastatin and amoxicillin-clavulanate; LTFU: lost to follow-up.

Among patients with regimen changes secondary to additional resistance detected by the tNGS results, eight of 31 (26%) were treated with rifampicin, isoniazid, pyrazinamide and ethambutol, and 23 of 31 were treated empirically with BDQ-containing DR-TB regimens. The final modifications resulted in 78% (18/23) of patients who were initially on bedaquiline-containing regimens being treated with individualised regimens without bedaquiline (Table 3). The CAC considered comorbidities, disease extent, and treatment progress when making these recommendations. The overall treatment success rate was 88%, patient fatality rate was 8%, and the lost to follow-up rate was 3%. This high treatment success rate has been mirrored by the programmatic DR-TB outcomes overall. Treatment success for all DR-TB patients nationally increased from 77.1% in 2019 to 86.3% in 2024, with a decline in deaths and treatment failure over the same period (Supplemental Table 6).

## Discussion

Our data describing the programmatic implementation and adoption of tNGS based diagnostics for clinical use in Eswatini highlight the potential clinical impact of introducing this technology for the diagnosis of drug-resistant TB and its feasibility in high-incidence, low-resource countries. The tNGS results not only led to regimen changes in 53% of the patients who underwent testing in the subset with complete clinical data but also highlighted the extent of the association between rifampicin resistance associated with the *rpoB* I491F mutation and mutations associated with bedaquiline and clofazimine resistance.

This association has been previously described in studies from Eswatini and neighbouring countries, including earlier work from our group^5,14^. However, our findings extend these observations by demonstrating the programmatic scale and clinical relevance of this resistance pattern during the routine implementation of sequencing within the national TB program. Within our cohort, 55% of RR-TB strains carried mutations associated with resistance to both bedaquiline and clofazimine, indicating that this co-occurrence represents a substantial proportion of circulating drug-resistant strains detected during routine care.

It is particularly concerning that most of these patients had never been previously exposed to bedaquiline. Among the 48 participants with these mutations and a documented history of prior TB treatment, only one had prior exposure to bedaquiline, suggesting the transmission of resistant strains rather than acquired resistance. This is further underlined by the fact that 85 out of the 87 BDQ-resistant strains had either a Rv0678 M146T (n = 69) or a Rv0678 N98D (n = 16) mutation, all of which were found in an MDR strain with an I491F mutation, thus linked with MDR strains with *rpoB* I491F mutations, which have already been described previously^5^. These findings underscore the importance of expanded genomic surveillance and the routine implementation of tNGS. tNGS-triggered therapeutic changes were common in our cohort, and treatment success was high, a finding which further supports the need to expand access to tNGS for people with DR-TB. Program data have also demonstrated continuous improvement in successful outcomes, potentially benefiting from individualised therapy facilitated by tNGS.

The increase of MDR Mtb strains with *rpoB* I491F mutations over time in Eswatini suggests a selective advantage by initial diagnostic escape linked with enhanced spread^5,6,8,14^. Overall, the prevalence of the epidemic *rpoB* I491F MDR Mtb strain among drug-resistant Mtb strains in the country doubled in the decade since its detection in 2009 and has likely expanded further since the last resistance survey in 2018^5–7^. This occurred despite the inclusion of phenotypic DST in the routine diagnostic algorithm for all patients with TB. This strategy was designed to mitigate the impact of the *rpoB* I491F strain, with evidence subsequently emerging of the inability of MGIT pDST to correctly classify strains with the *rpoB* I491F mutation as RR using the WHO-recommended critical concentration^5^.

tNGS can have an important impact on the care of patients infected with drug-resistant Mtb strains with the *rpoB* I491F mutation or other mutations that are either located outside the 80 bp hotspot region in *rpoB* (interrogated by most rapid molecular DST assays) or those leading to low-level resistance. We found that 35%–47% of patients in our cohort would have received a correct regimen based on the results of routine tests. While the impact of tNGS in Eswatini may be particularly profound, recent data from South Africa, Botswana, and Mozambique also demonstrate the emergence of Mtb strains with *rpoB* I491F mutations^7,15^, strongly arguing for the urgent need for the programmatic introduction of tNGS in other high-incidence countries in the region. This also demonstrates the urgent need to expand the coverage of *rpoB* probes to detect the I491F mutation on GeneXpert machines and other molecular DST assays.

The detection of mutations in the Rv0678 gene, associated with bedaquiline and clofazimine resistance, is an important finding from the sequencing program^7^. This data coincides with increasing certainty that these mutations result in increased minimum inhibitory concentrations for bedaquiline^10,16,17^. However, it is important to note that, based on the current version of the WHO resistance catalogue, the sensitivity of Deeplex tNGS to detect bedaquiline resistance among rifampicin-resistant samples is relatively low (67.9%) compared to its performance with other drugs, such as isoniazid, rifampicin, levofloxacin, moxifloxacin, ethambutol, and streptomycin, for which the pooled sensitivity is ≥93%^16^. As a result, these data might underestimate the actual number of bedaquiline and clofazimine-resistant strains. The detection of bedaquiline resistance in Eswatini is consistent with the emerging bedaquiline resistance among patients in South Africa and Mozambique^7,18^ and is the highest bedaquiline resistance rate reported to date. It is unclear why this mutation is so strongly associated with the *rpoB* I491F epidemic strain, but the transmission success of *rpoB* I491F strains seems to have contributed to the increase in bedaquiline resistance in the country. The observed discordance between the tNGS and pDST results for bedaquiline may be attributable to the use of MGIT pDST at a single critical concentration. The absence of MIC testing and the use of MGIT pDST are limitations of this study. In MDR strains from Eswatini, the main Rv0678 mutations are M146T and N98D, which have been shown to lead to a moderate bedaquiline MIC increase in our previous work^5,13^. These results are in line with other Rv0678 mutations which render resistance detection by MGIT pDST tested with a single critical concentration difficult.

Infection with MDR/RR Mtb strains with Rv0678 gene mutations has been linked to worse treatment outcomes^10,19^. This has important implications for the global adoption of bedaquiline-pretomanid-linezolid-(moxifloxacin) as first-line regimens for MDR/RR TB, which may be less effective when bedaquiline resistance is present^19,20^. Bedaquiline-pretomanid-linezolid-(moxifloxacin) was recently adopted in Eswatini as a first-line regimen for rifampicin-resistant TB, a decision that is now being reconsidered based on the findings from programmatic sequencing. This again highlights the need for tests that provide rapid detection of bedaquiline resistance^12^ and underlines that, when possible, rapid molecular resistance tests should be released in coordination with the launch of new treatment regimens.

The resistance patterns observed in Eswatini also raise questions about the WHO classification of patients with drug-resistant TB^21^. Currently, pre-extensively drug-resistant (pre-XDR) TB is defined as resistance to rifampicin (with or without resistance to isoniazid) and resistance to fluoroquinolones. Extensively drug-resistant (XDR) TB is defined as pre-XDR with additional resistance to bedaquiline and/or linezolid. In Eswatini, 50% (80/159) of MDR/RR *Mtb* strains had additional bedaquiline resistance without resistance to fluoroquinolones; hence, they cannot be classified as XDR or pre-XDR. Similar patterns have also been observed for MDR/RR *Mtb* strains from Mozambique^7^. Given the introduction of bedaquiline-pretomanid-linezolid-(moxifloxacin) as a first-line regimen for MDR TB and pre-XDR drug-resistant TB, this pattern of resistance is arguably more clinically impactful than pre-XDR drug-resistant TB as currently defined. This pattern of resistance suggests the need for the reclassification of drug-resistant TB categories by the WHO.

This report provides evidence that tNGS can have enormous implications for the composition of the resulting drug regimens for patients affected by drug-resistant TB. The introduction of tNGS in Eswatini was achieved through a well-coordinated approach that integrated and aligned the resources of the Ministry of Health programs and partners and was accompanied by clinical training and support from internal and external experts.

The programmatic introduction of tNGS was not without substantial challenges, but experience with establishing the technology elsewhere in the region for surveillance and research informed the program^22^. Support for the adoption of tNGS, in the absence of a WHO recommendation, was generated within the Ministry of Health through the clear dissemination of the national drug resistance survey results and engagement with WHO offices at the local and international levels. At the NTRL, implementation required structural renovations to reduce the risk of amplicon contamination impacting the sequencing results. The largest technical challenge, however, was the failed sequencing runs. This was addressed through extensive training, troubleshooting and commodity replacement. In the absence of established procurement networks, it is likely that some of the flow cells were damaged during transport and storage, resulting in substantial challenges to the project and delaying implementation. Automation of tNGS and cost estimations are needed to speed up the integration into programmatic diagnostic algorithms more broadly in low-resource settings. Given some of these challenges, we do not have precise estimates for tNGS time to result or time to treatment regimen change. Sustainability remains a major challenge but was addressed throughout implementation through MOH leadership. With WHO recommendations for tNGS use and support from international funders, it is also expected that prices will continue to improve, as was seen with Xpert rollout. Even at current prices, the cost-effectiveness of tNGS has been demonstrated in similar contexts^23^. The need for phenotypic drug susceptibility testing will also need to be considered, as replacement rather than supplementation with tNGS is likely to be more cost effective^23^. Additional limitations of this report are typical of programmatic reports and include incomplete data, variability in the timing of result reporting, and an imperfect programmatic reference standard with culture and pDST. Furthermore, as is common in programmatic DR-TB cohorts from high-burden settings, prior treatment history was incompletely captured in the surveillance database, limiting our ability to distinguish new from re-treatment cases across the full cohort. Lastly, not all people diagnosed with TB underwent tNGS. The population chosen for tNGS by the diagnostic algorithm may not be indicative of how widespread this problem is (which can only be known through comprehensive active surveillance or formal drug resistance surveys). The diagnostic algorithm used to indicate the need for sequencing was dependent on the detection of isoniazid or rifampicin resistance. As such, it is possible that we are underestimating the prevalence of Mtb strains with the *rpoB* I491F mutation or that we are overestimating the prevalence if strains detected by Xpert were less likely to be sent to the NTRL.

The decision regarding where to place tNGS in the diagnostic algorithm considered cost and sequencing capacity. This was also based on data from a prior National Drug Resistance Survey, which found that approximately 94% of strains with the *rpoB* I491F mutation also had isoniazid resistance. Furthermore, as outlined above, tNGS may underestimate bedaquiline resistance, especially as the current assay only detects mutations in Rv0678 and not in other targets such as atpE. Similarly, there are still limited data on the impact of some Rv0678 mutations on bedaquiline MIC levels, and we cannot exclude some overestimation of clinical resistance attributable to genotypic mutations.

In conclusions, the implementation of tNGS in Eswatini with the integration of sequencing results into clinical care was successful, largely due to the strong network of drug-resistant TB physicians and nurses’ eager for results that detected the actual drug resistance patterns that they were treating. Overall, targeted sequencing resulted in treatment adaptation in 53% of patients in this cohort. Our study shows that the introduction of programmatic sequencing is feasible for programs and increases the use of effective treatment for patients. tNGS should enable physicians to design an effective treatment regimen for each patient and may prevent the selection of drug-resistant strains of Mtb by TB program policies that follow a one-regimen-fits-all solution. Our data also revealed the highest bedaquiline/clofazimine resistance mutation rate among RR Mtb strains observed so far in a programmatic cohort. Both the high rate of rifampicin resistance due to *rpoB* I491F strains and the high bedaquiline/clofazimine mutation resistance rate in Eswatini emphasise the urgent need to scale up diagnostics for drug-resistant TB patients. tNGS can help close the diagnostic gap that is currently contributing to treatment failure, drug resistance amplification, and ongoing transmission of drug-resistant TB strains.

## Methods

### Ethics and study approvals

This study complied with all relevant ethical regulations. The protocol for programmatic implementation was approved by the Eswatini Health and Human Research Board (IRB #FWA00026661) and the Baylor College of Medicine Institutional Review Board, Houston, Texas, USA (H-49309). Informed consent was obtained from all the participants. Diagnostic testing and treatment were performed within the routine programmatic care. Sex was recorded in routine programmatic records obtained through clinical self-reporting; gender was not separately collected, and no sex-stratified analyses were pre-specified for this implementation study. The results were disaggregated appropriately, where data for sex/gender were available (median age 37; 65% male). Participant compensation was not provided as it was integrated into routine programmatic care.

### Study setting and design

In 2019, the Eswatini Ministry of Health, in consultation with local partners and drug-resistant tuberculosis experts, decided to introduce tNGS testing. Since this technology was not yet recommended by the WHO for routine use, the country opted to implement tNGS as a project with engagement from the WHO’s local and international offices. A protocol for programmatic implementation was developed and approved by the Eswatini Health and Human Research Board (IRB #FWA00026661) and Baylor College of Medicine Institutional Review Board, Houston, Texas, USA (H-49309), and used as a framework for laboratory and clinical implementation.

### Program population

tNGS was performed on samples received at the National Tuberculosis Reference Laboratory that met any of the following criteria: 1. At least one resistant result to isoniazid and/or rifampicin identified by an initial genotypic diagnostic tests available in Eswatini (Xpert Ultra, Xpert XDR, LPA (Hain Lifescience GmbH, Nehren, Germany); 2. At least one resistant result indicating resistance to either isoniazid or rifampicin using MGIT pDST; or 3. or suspected treatment failure, as requested by the treating physician. A total of 442 individuals were enrolled in the national TB program from June 2021-December to 2024 with confirmed and unconfirmed drug resistance based on routine diagnostic testing.

### Laboratory methods

Consistent with the Eswatini national guidelines and routine practise, each patient provided two sputum specimens. One was tested using Xpert Ultra in accordance with the manufacturer’s instructions at peripheral facilities. From the Xpert Ultra confirmed tuberculosis patients, the second specimen was further tested by LPA and culture using the MGIT 960 system (Becton Dickinson, Franklin Lakes, USA) at the National Tuberculosis Reference Laboratory (NTRL) in Mbabane, Eswatini, as instructed by the manufacturers. In short, specimens were decontaminated using N-acetyl-L-cysteine/NAOH (1% NaOH final concentration) and resuspended after centrifugation in 2 ml phosphate buffer. Subsequently, 0.5 ml of the suspension was inoculated into MGIT tube media, while 0.5 ml was used for extraction of the genomic DNA directly from sputum. All positive cultures were tested for susceptibility to first-line drugs, rifampicin, isoniazid, and ethambutol, using the WHO-recommended critical concentrations (the recommended critical concentration for rifampicin is 0.5 ug/mL). Isoniazid-resistant or MDR/RR strains were additionally tested for susceptibility at the recommended critical concentrations for bedaquiline, clofazimine, linezolid, delamanid, and levofloxacin/moxifloxacin (the WHO-recommended critical concentrations were used for all drugs, specifically 1.0 ug/mL for bedaquiline).

### Sequencing and data analysis

The Deeplex® Myc-TB assay (GenoScreen, Lille, France) was used for tNGS testing. The assay targets full sequences (i.e., coding sequence plus part of the promoter region) or the most relevant regions of 18 drug resistance-associated genes (*rpoB, ahpC, fabG1, katG, inhA, pncA, embB, gyrA, gyrB, rrs, eis, tlyA, gidB rpsL, ethA, rv0678, rrl, rplC*), combined with genomic targets for mycobacterial species identification *(hsp65)* and Mtb strain genotyping (CRISPR locus)^24,25^. For Deeplex® Myc-TB, DNA extractions were performed for routine LPA using the GenoLyse® extraction kit (Hain Lifescience GmbH, Nehren, Germany), followed by ethanol precipitation. After Deeplex® Myc-TB amplification was performed as instructed by the manufacturer, amplicon libraries were prepared using a modified Illumina Nextera-based protocol^26–28^ and sequenced with 150 bp paired-end reads using an iSeq 100 instrument (Illumina, San Diego, California, USA). Mutation detection was performed using the bioinformatics pipeline on the Deeplex® Myc-TB web application with the setting “Deeplex Myc-TB V3_0_1 - Extended catalogue”. The detected variants were automatically associated with drug resistance or susceptibility or phylogenetic lineage based on the selected catalogue. If the variants were uncharacterized, polymorphisms were further interpreted using a curated mutation catalogue employed at the Research Center Borstel (v2022-11-22), as described previously^24,25^.

### Programmatic treatment

A subset of patients with sequencing results who had clinical information submitted by clinicians to the Clinical Advisory Committee (CAC) were reviewed. The CAC reviewed all drug susceptibility test results, including tNGS results, in conjunction with clinical information on the patient provided by the clinician. In consultation with the National TB Control Program (NTCP) and CAC chair, recommendations for treatment adjustments were provided to the treating clinicians (Fig. 1C). Their treatment outcomes were later followed through programmatic structures and documented in a sequencing database.

### Statistical analysis and role of the funder

Data obtained with the Deeplex®-MycTB assay were compared descriptively and visualized using R (version 4.5.1), with data obtained from the other tests, including LPA, Xpert Ultra, and MGIT pDST of grown Mtb strains. The tNGS data were further compared descriptively in a subset of patients with clinical information from the chronic care and treatment registers shared by the drug-resistant tuberculosis clinical team using a standardised template developed by the NTCP, EHLS, and implementing partners.

The funder of the study had no role in the study design, data collection, data analysis, data interpretation, or writing of the report.

### Reporting summary

Further information on research design is available in the Nature Portfolio Reporting Summary linked to this article.

## Supplementary information

Supplementary Information

Peer Review file

Description of Additional Supplementary Files

Supplementary Data 1

Reporting Summary

## Data Availability

The targeted next-generation sequencing data generated in this study have been deposited in the European Nucleotide Archive (ENA) database https://www.ebi.ac.uk/ena/browser/home under accession codes listed in Supplementary Data 1. Clinical and epidemiological data are available under restricted access due to patient data privacy laws. Access to individual participant data underlying the results in the text, tables, and figures can be obtained by submitting a methodologically sound protocol (registered on PROSPERO) for an individual patient meta-analysis. Requests must be submitted to Dr. Debrah Vambe (Debrah.Vambe@bcm.edu) and will be reviewed by the Eswatini NTCP. Access will be granted contingent on the execution of the appropriate data access agreements.

## Code availability

Not applicable. No custom code essential for reproducing the principal findings was generated in this study. Sequencing analysis used established assay-specific pipelines and validated bioinformatic workflows, as described in the Methods section.
