# Supplementary material for: Targeted next-generation sequencing implementation in Eswatini identifies rifampicin and bedaquiline resistance undetected by routine diagnostic testing
Source: Nat Commun. 2026 Jun 11;17:8227. doi: 10.1038/s41467-026-73551-w (PMC13462916; doi:10.1038/s41467-026-73551-w)
Supplement: Supplementary file 1 [file 41467_2026_73551_MOESM1_ESM.pdf]

Supplementary Information

Targeted Next-Generation Sequencing Implementation in Eswatini Identifies Rifampicin and Bedaquiline Resistance Undetected by Routine Diagnostic Testing

Author List

*Debrah Vambe[1,2], *Alexander Kay[1,2], *Mangaliso Ziyane[1,3,4], Mdigo Thunzini[1,3], Sein Sein Thi[5], Leonardo de Araujo[6,7], Viola Dreyer[6,7], Tanja Niemann[6,7], Busizwe Sibandze[3], Sijabu Masina[5], Andrea Maurizio Cabibbe[8], Jennifer Furin[9], Tafadzwa Mazuruse[10], Lindokuhle Dlamini[11], Thulani Jele[5], Bheki Mamba[5] Gugu Maphalala[3], Makhosazana Dlamini[1], Mbongeni Dube[1], Nomthandazo Lukhele[12], Daniela Maria Cirillo[8], Anna Mandalakas[2,13,14], Christoph Lange[2,13,14,15], Sphiwe Ngwenya[5], ^Christian Utpatel[6,7], ^Sindisiwe Dlamini,[3] ^Stefan Niemann[6,7,16,17]

Table of Contents

Supplementary Standard Operating Procedure (SOP) 2

Procedures for incorporation of targeted sequencing results into DR-TB clinical care (National TB Control Program and National TB Reference Laboratory) 2

Supplementary Table 1. Key drug resistance by test within the complete cohort. . 3

Supplementary Table 2. tNGS mutations identified for rifampicin and bedaquiline associated with resistance. 3

Supplementary Table 3. WHO resistance classification of matched strains by tNGS vs. routine diagnostics 4

Supplementary Table 4. Paired tNGS vs. pDST results for bedaquiline resistance (N = 84). 4

Supplementary Table 5A-F. Contingency tables demonstrating agreement and disagreement in WHO drug-resistance classifications by routine diagnostics compared to tNGS. 5

A. tNGS classification compared to Xpert Ultra (N = 133) 5

B. tNGS classification compared to LPA (N = 192) 5

C. tNGS classification compared to LPA restricted to strains with first- and second-line LPA testing (N = 144) 5

D. tNGS classification compared to MGIT pDST (N = 205) 6

E. tNGS classification compared to MGIT pDST restricted to strains with results for fluoroquinolones and bedaquiline (N = 76) 6

F. tNGS classification compared to composite routine diagnostics (N = 234) 6

Supplementary Table 6. Programmatic treatment outcomes before and after tNGS implementation. 7

Supplementary Figure 1. Drug-resistance reclassification by tNGS among the 76 patients with complete first- and second-line MGIT pDST results 8

Supplementary Data 1. Sample-level European Nucleotide Archive (ENA) accession codes 9

Supplementary Standard Operating Procedure (SOP).

Procedures for incorporation of targeted sequencing results into DR-TB clinical care (National TB Control Program and National TB Reference Laboratory)

This standard operating procedure (SOP) describes the workflow for incorporation of targeted next-generation sequencing (tNGS) results into the programmatic management of drug-resistant tuberculosis (DR-TB) in Eswatini, jointly implemented by the National Tuberculosis Control Program (NTCP) and the National Tuberculosis Reference Laboratory (NTRL) with support from implementing partners.

Scope: Sputum specimens received at the NTRL from patients meeting at least one of the following criteria are eligible for tNGS: (i) detection of resistance to isoniazid and/or rifampicin by Xpert MTB/RIF Ultra, Xpert XDR, or line probe assay (LPA); (ii) phenotypic resistance to isoniazid or rifampicin by MGIT pDST; or (iii) suspected treatment failure as requested by the treating clinician.

Sample handling and laboratory workflow: Specimens are decontaminated using N-acetyl-L-cysteine/NaOH (1% NaOH final concentration) and resuspended in phosphate buffer. DNA is extracted directly from sputum using the GenoLyse kit (Hain Lifescience) followed by ethanol precipitation, and amplification is performed using Deeplex Myc-TB (GenoScreen). Amplicon libraries are prepared with a modified Illumina Nextera-based protocol and sequenced (150 bp paired-end) on the iSeq 100 platform. Variant calling and resistance classification use the Deeplex Myc-TB extended catalogue (V3_0_1) supplemented with the Research Center Borstel curated catalogue (v2022-11-22) for rare or uncharacterised mutations.

Reporting: Upon receipt of tNGS report and other routine tests results, they are released to the treating clinician, and the Clinical Advisory Committee (CAC) chair through a secure shared workspace, with parallel entry into the national TB surveillance database. The CAC chair requests the treating clinician to fill in the clinical information for the patient using the patient report form (PRF) and submit to Clinician advisory committee.

Clinical Advisory Committee (CAC): The CAC reviews all tNGS reports together with the patient report form (PRF) submitted by the treating clinician. The CAC issues written treatment recommendations within 5 working days of receiving a complete PRF. Recommendations consider comorbidities, prior TB drug exposure, fluoroquinolone and bedaquiline resistance patterns, and disease extent. Routine bi-weekly DR-TB clinical meetings provide a forum for de-identified case discussion and ongoing clinician training.

Quality assurance and monitoring: NTCP and NTRL jointly monitor sequencing run success rates, turn-around times, and concordance with phenotypic DST through quarterly indicators. The SOP is reviewed annually or whenever the WHO catalogue of mutations or national treatment guidelines are updated.

Supplementary Table 1. Key drug resistance by test within the complete cohort.

Drug / Test	Total N	Resistant	Susceptible	Indeterminate / Invalid*	Data Not Available
Isoniazid					
Xpert XDR	10	10 (100%)	0	0	224
Line Probe Assay	192	155 (80.7%)	36 (18.8%)	1 (0.5%)	42
MGIT pDST	212	178 (83.9%)	21 (10.0%)	13 (6.1%)	22
tNGS	234	200 (85.5%)	34 (14.5%)	0	0
Rifampicin					
Xpert Ultra	133	43 (32.3%)	89 (66.9%)	1 (0.75%)	101
Line Probe Assay	190	37 (19.5%)	152 (80.0%)	1 (0.5%)	44
MGIT pDST	214	42 (19.6%)	161 (75.2%)	11 (5.1%)	20
tNGS	234	159 (67.9%)	75 (32.1%)	0	0
Fluoroquinolone					
Xpert XDR	10	1 (10.0%)	9 (90.0%)	0	224
Line Probe Assay	149	5 (3.4%)	143 (96.0%)	1 (0.6%)	85
MGIT pDST	129	4 (3.1%)	118 (91.5%)	7 (5.4%)	105
tNGS	234	11 (4.7%)	223 (95.3%)	0	0
Bedaquiline					
MGIT pDST	102	5 (4.9%)	79 (77.5%)	18 (17.6%)	132
tNGS	234	87 (37.2%)	147 (62.8%)	0	0

**Invalid = contamination or under-inoculation errors after the test was repeated once.*

Supplementary Table 2. tNGS mutations identified for rifampicin and bedaquiline associated with resistance.

Resistance category	Mutation	N (%)
Rifampicin Resistance (N = 159)	I491F	96 (58.8%)
	H445D | I491F	1 (0.6%)
	S450L | I491F	4 (2.4%)
	S450L	36 (24.1%)
	S450L | G426S	1 (0.6%)
	H445D	6 (3.5%)
	H445L	6 (3.5%)
	H445Y	3 (1.8%)
	H445C	2 (1.2%)
	D435V	1 (0.6%)
	H445R	1 (0.6%)
	H445R | S450L	1 (0.6%)
	Q432L	1 (0.6%)
Bedaquiline Resistance (N = 87)	Frameshift*	2 (2.2%)
	Frameshift | M146T	1 (1.1%)
	M146T	67 (77.0%)
	N98D	16 (18.4%)
	S53A | M146T	1 (1.1%)

**86/87 BDQ-resistance mutations were associated with an I491F mutation; one frameshift mutation was identified in a strain with an isolated S450L mutation.*

Supplementary Table 3. WHO resistance classification of matched strains by tNGS vs. routine diagnostics.

Routine diagnostic classification (Xpert Ultra, LPA, Xpert XDR, MGIT pDST)	tNGS classification	N (Total = 234)
DS	DS	20
DS	INH-R	1
DS	MDR	1
INH-R	DS	5
INH-R	INH-R	44
INH-R	MDR	18
INH-R	MDR (BDQ-R)	78
INH-R	Pre-XDR	2
INH-R	XDR	6
MDR	INH-R	3
MDR	MDR	36
MDR	MDR (BDQ-R)	2
MDR	Pre-XDR	1
MDR	RR	5
MDR (BDQ-R)	MDR	1
Pre-XDR	Pre-XDR	1
RR	DS	2
RR	MDR	5
RR	RR	2
XDR	XDR	1

Supplementary Table 4. Paired tNGS vs. pDST results for bedaquiline resistance (N = 84).

MGIT pDST	tNGS Resistant	tNGS Susceptible
Resistant	4	1
Susceptible	22	57

Supplementary Table 5A-F. Contingency tables demonstrating agreement and disagreement in WHO drug-resistance classifications by routine diagnostics compared to tNGS.

A. tNGS classification compared to Xpert Ultra (N = 133)

Xpert Ultra	DS	INH-R	MDR	MDR (BDQ-R)	Pre-XDR	RR	XDR
DS	8	27	9	42	0	0	4
RR	1	2	34	1	1	4	0

Agreement: 46/133 = 0.346.

B. tNGS classification compared to LPA (N = 192)

LPA	DS	INH-R	MDR	MDR (BDQ-R)	Pre-XDR	RR	XDR
DS	19	2	1	0	0	0	0
INH-R	1	38	18	68	2	1	5
MDR	0	1	18	2	0	0	0
Pre-XDR	0	0	0	0	1	0	0
RR	0	0	9	0	0	6	0

Agreement: 82/192 = 0.427; inclusive of MDR/RR: 91/192 = 0.474.

C. tNGS classification compared to LPA restricted to strains with first- and second-line LPA testing (N = 144)

LPA	DS	INH-R	MDR	MDR (BDQ-R)	Pre-XDR	RR	XDR
DS	2	0	0	0	0	0	0
INH-R	1	33	10	58	0	1	5
MDR	0	0	16	2	0	0	0
Pre-XDR	0	0	0	0	1	0	0
RR	0	0	9	0	0	6	0

Agreement: 58/144 = 0.403; inclusive of MDR/RR: 67/144 = 0.465.

D. tNGS classification compared to MGIT pDST (N = 205)

MGIT	DS	INH-R	MDR	MDR (BDQ-R)	Pre-XDR	RR	XDR
DS	19	3	1	1	0	0	0
INH-R	5	38	17	71	2	1	5
MDR	0	2	29	1	1	4	0
Pre-XDR	0	0	0	0	1	0	0
RR	1	0	1	0	0	1	0
XDR	0	0	0	0	0	0	1

Agreement: 89/205 = 0.434; inclusive of MDR/RR: 94/205 = 0.459.

E. tNGS classification compared to MGIT pDST restricted to strains with results for fluoroquinolones and bedaquiline (N = 76)

MGIT	DS	INH-R	MDR	MDR (BDQ-R)	Pre-XDR	RR	XDR
DS	0	1	0	0	0	0	0
INH-R	1	19	5	23	0	0	1
MDR	0	1	18	0	0	2	0
Pre-XDR	0	0	0	0	1	0	0
RR	1	0	1	0	0	1	0
XDR	0	0	0	0	0	0	1

Agreement: 40/76 = 0.526; inclusive of MDR/RR: 43/76 = 0.566.

F. tNGS classification compared to composite routine diagnostics (N = 234)

Composite	DS	INH-R	MDR	MDR (BDQ-R)	Pre-XDR	RR	XDR
DS	20	1	1	0	0	0	0
INH-R	5	44	18	78	2	0	6
MDR	0	3	36	2	1	5	0
MDR (BDQ-R)	0	0	1	0	0	0	0
Pre-XDR	0	0	0	0	1	0	0
RR	2	0	5	0	0	2	0
XDR	0	0	0	0	0	0	1

Agreement: 104/234 = 0.444; inclusive of MDR/RR: 114/234 = 0.487.

Supplementary Table 6. Programmatic treatment outcomes before and after tNGS implementation.

Phase	Year	Treatment success (cured + completed)	Cured	Completed	Died	LTFU	Failed
Pre-implementation	2019	77.1%	74.7%	2.5%	15.7%	2.2%	4.7%
	2020	81.1%	78.0%	3.0%	11.1%	2.7%	5.1%
Post-implementation	2021	80.4%	76.8%	3.6%	11.1%	5.2%	3.2%
	2022	75.2%	69.9%	5.2%	12.4%	6.5%	5.9%
	2023	84.2%	62.4%	21.8%	7.9%	4.2%	3.6%
	2024	86.3%	18.3%*	68.0%*	9.2%	3.9%	0.7%

**Low cure rate in 2024 reflects culture reagent stock-outs; the rise in "Completed" outcomes reflects difficulty assigning a "Cured" outcome under WHO criteria.*

Supplementary Figure 1. Drug-resistance reclassification by tNGS among the 76 patients with complete first- and second-line MGIT pDST results.
Seventy-six participants had MGIT pDST testing for isoniazid, rifampicin, fluoroquinolones, and bedaquiline. The left pillar of the alluvial plot shows the baseline WHO tuberculosis drug-resistance classification (drug-susceptible TB defined as the absence of resistance to isoniazid and rifampicin; isoniazid-resistant TB; rifampicin-resistant TB; multidrug-resistant TB; pre-extensively drug-resistant TB; extensively drug-resistant TB) using results for isoniazid, rifampicin, fluoroquinolones, bedaquiline, and linezolid based on MGIT pDST. The right pillar shows the drug-resistance classification after incorporation of tNGS results; all 76 participants had a valid tNGS result. Abbreviations: DS, drug-susceptible; INH-R, isoniazid-resistant; MDR, multidrug-resistant; RR, rifampicin-resistant without evidence of other drug resistance; MDR (BDQ-R), MDR with bedaquiline resistance; Pre-XDR, MDR with fluoroquinolone resistance; XDR, Pre-XDR with additional bedaquiline or linezolid resistance.

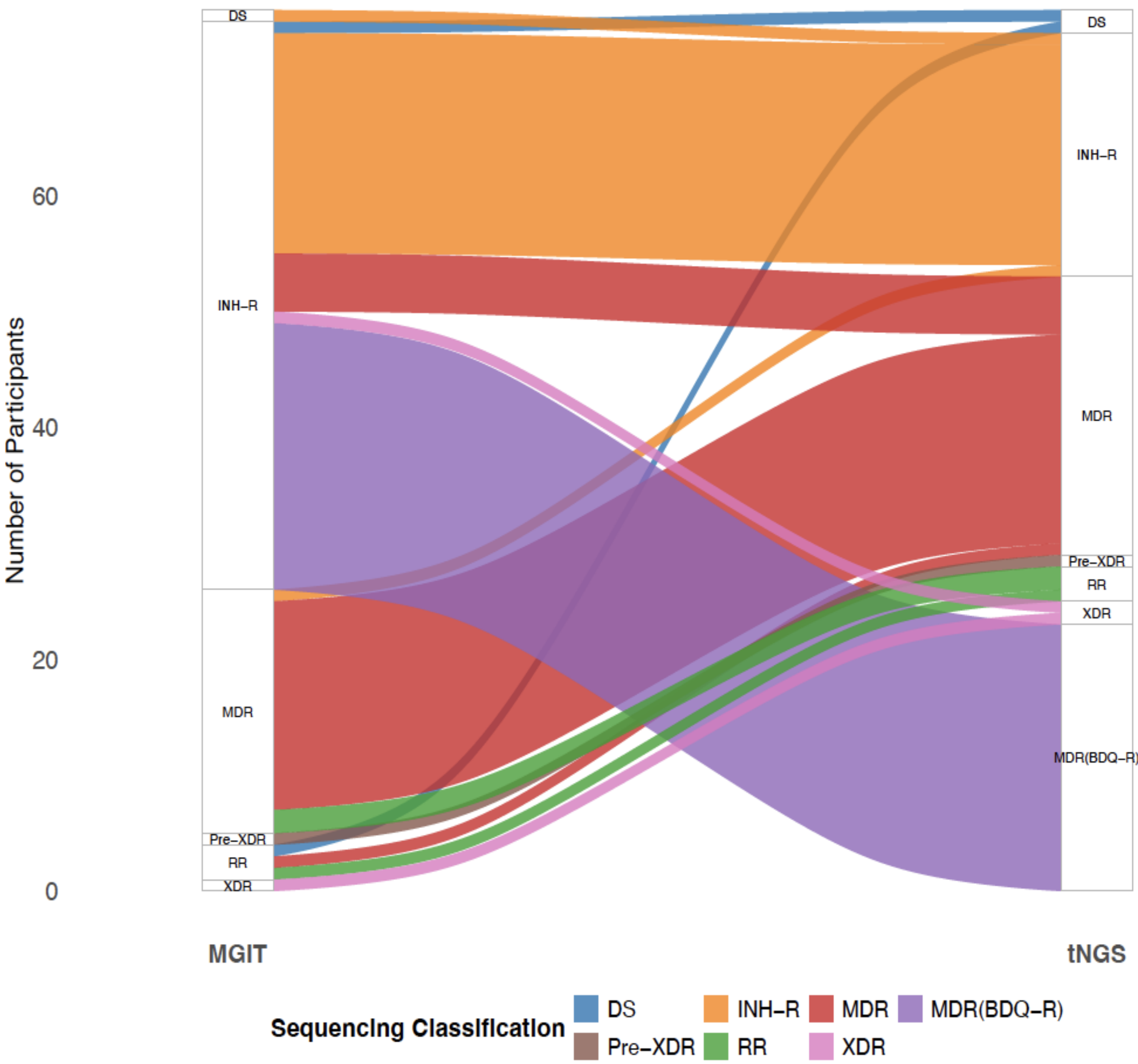

Supplementary Data 1. Sample-level European Nucleotide Archive (ENA) accession codes

Sample-level European Nucleotide Archive (ENA) accession codes and per-drug resistance calls for all 234 sequenced samples. Provided as a separate Excel (.xlsx) file. Each row represents one sample. Columns: SampleID, PatientID, ENA_ACCESSION_NUMBER, Year, Sequencing_result_acceptability, Composite_target_coverage_breadth (%), Average_coverage_depth (x), Species best match, RIF, INH, PZA, EMB, SM, FQ, KAN, AMI, CAP, ETH, LIN, BDQ, CFZ (0 = susceptible; 1 = resistant; "" = no result), and Resistance_Category. Raw sequencing reads have been deposited in the European Nucleotide Archive under the accession codes listed.
