# Supplementary material for: Targeted next-generation sequencing implementation in Eswatini identifies rifampicin and bedaquiline resistance undetected by routine diagnostic testing
Source: Nat Commun. 2026 Jun 11;17:8227. doi: 10.1038/s41467-026-73551-w (PMC13462916; doi:10.1038/s41467-026-73551-w)
Supplement: Supplementary file 2 [file 41467_2026_73551_MOESM2_ESM.pdf]

nature portfolio

Peer Review File

Targeted Next-Generation Sequencing Implementation in Eswatini Identifies Rifampicin and Bedaquiline Resistance Undetected by Routine Diagnostic Testing

Corresponding Author: Professor Stefan Niemann

This file contains all reviewer reports in order by version, followed by all author rebuttals in order by version.

Attachments originally included by the reviewers as part of their assessment can be found at the end of this file.

Version 0:

Reviewer comments:

Reviewer #1

(Remarks to the Author)
This manuscript describes the process of and early experience with the programmatic introduction and implementation of tNGS in Eswatini in response to the rapidly increasing prevalence of rifampicin-resistant TB driven by an epidemic strain with a “diagnostic escape” strain. The rpoB I491F mutation that defines rifampicin resistance in the outbreak strain is not detected by Xpert and LPA and often phenotypic DST; and, as previously reported, an alarming association with bedaquiline resistance has emerged in this strain. The results for the first 37 patient samples submitted for tNGS due to isoniazid- and/or rifampicin-resistance or inadequate treatment response are presented. The rpoB I491F mutation was identified in nearly half of the samples and nearly two-thirds of MDR isolates. Bedaquiline resistance was demonstrated in two-thirds of the isolates with the rpoB I491F mutation, in-line with recent reports related to this outbreak. No isolate was resistant to both bedaquiline and a fluoroquinolone. Overall, 73% of patients underwent a regimen change based on the tNGS results, some before and some after recommendation by the CAC. The authors conclude that tNGS is essential to detect I491F “diagnostic escape” strains and resistance to new TB drugs used for MDR-TB treatment, and has the potential to improve the management of drug-resistant TB in high-burden settings like Eswatini.

Major comments:
This report clearly demonstrates the feasibility (with sufficient investment) and potential impact of tNGS in the context of an MDR-TB outbreak driven by a strain capable of diagnostic escape. As such a test case, the report is timely and likely to be impactful. Some additional major and minor comments are provided below.

It would be helpful to know the average turnaround time for the tNGS results and CAC recommendations to be returned to the clinicians. At what point in treatment were the regimen changes based on tNGS made?

Please explain how indeterminate RIF phenotypic DST results were defined and whether an indeterminate result was associated with the presence of the I491F mutation.

If phenotypic bedaquiline DST or MIC testing was performed on some or all of the isolates, those results would be welcome.

A perspective on the perceived sustainability of the program in Eswatini would be welcome in the discussion.

Publication of CAC job aids and SOPs in supplemental material would be valuable.

Minor comments

Introduction
Paragraph 2, last sentence: reference needed for this statement
Paragraph 3, first sentence: the claim that the I491F mutation is not detected by phenotypic assays should be more nuanced

Materials and methods
Line 2: “endorsed” or “recommended” by WHO may be more appropriate, as WHO does not “approve” products per se
Programmatic treatment, first sentence: CAC should be spelled out with first use

Results
Clinical integration of tNGS, first line: what is meant by “sensitization”?
Initial tNGS results, second paragraph, line 8: “20% (1 of 5) as drug-resistant…” should read “20% (1 of 5) as MDR…”?
Penultimate paragraph: please explain why report forms were not received from 7 of 37 patients and why a recommendation was provided by the CAC for one patient. Of the 15 patients in which a change was recommended, how many actually had a change made in their regimen?

Discussion
Paragraph 3, line 5: suggest to replace “ineffective due to” with “expected to be ineffective due to” or “prescribed despite” or something similar to emphasize that not all mutations confer absolute resistance and effectiveness was not measured

Please explain why the RIF susceptibility results in Fig 2A and Fig 3A-C do not align in terms of the proportions that are susceptible, resistant, indeterminate and missing.

Fig 1B legend: please describe the meaning of the blue vs. orange boxes

Fig 3 legend: what is the “Bottom Row” that is referred to? Did the authors intend to refer to Left and Right columns instead of Top and Bottom rows, respectively? Yellow shading in the Left columns is not defined in the legend. The light blue and dark blue shading described in the legend is not apparent in the figure.

Reviewer #2

(Remarks to the Author)
Vambe et al. present a pilot study exploring the implementation of targeted next-generation sequencing (tNGS) as a diagnostic tool for drug resistance in Eswatini. The study addresses a critical issue: the detection of the rpoB_I491F mutation, which confers rifampicin resistance and is highly prevalent in Eswatini as described in many previous publications. This mutation is undetectable by common frontline diagnostic tests such as those used in Eswatini (Xpert Ultra, LPA and pDST). Thus, the implementation of a diagnostic tool capable of identifying this and other undetected mutations, by the frontline tools, is essential. While the study addresses an important topic, the title does not fully reflect the scope or findings, as the small sample size limits a comprehensive evaluation of the clinical impact of tNGS.
Furthermore, the abstract does not clearly state the primary objective of the study, and the conclusion regarding the alarming situation of bedaquiline resistance is not supported by the data. Specifically, only 12 strains harboring bedaquiline resistance were identified in a dataset of 37 drug-resistant samples. Consequently, the authors cannot extrapolate these results to claim that bedaquiline resistance is an alarming issue in the country, as the study lacks a population-based design.
The study analyzed 37 samples, representing only 27% of the patients (119) recruited and approximately 15% of the MDR/RR cases (~240) estimated in Eswatini according to WHO’s 2021-2022 report. This small sample size limits the generalizability of the findings. Consequently, conclusions about mutation prevalence should be drawn cautiously. For instance, statements such as “The extraordinarily high rate of potential bedaquiline and clofazimine resistance is alarming” should be revised. These conclusions, based on a limited dataset, carry significant public health implications and risk overstating the findings. The manuscript primarily presents descriptive results as percentages based on a small sampling, without any statistical analysis or indicators of implementation success.
The mutation analysis is not sufficiently detailed. It is unclear which mutations are missed by each diagnostic test (it can be assumed that the 17 cases with rpoB_I491F are missed by XpertUltra, but an additional case is missing) and how rifampicin resistance mutations, such as rpoB_I491F, co-occur with mutations conferring resistance to other drugs like bedaquiline.
Furthermore, the authors should provide additional context for including Rv0678_N98D as a resistance-associated mutation, given that the WHO mutation catalogue classifies it as Uncertain (WHO-UCN-TB-2023.7).
The manuscript needs a more comprehensive discussion of the limitations of tNGS, particularly regarding its sensitivity for detecting mutations associated with bedaquiline resistance, which is estimated at only ~30% by the WHO catalogue.
Additionally, the selection of samples pre-identified as resistant to isoniazid (INH) and/or rifampicin (RIF) introduces bias, as rpoB_I491F—a critical mutation in Eswatini—is not detectable by the methods used. This could lead to an underestimation of RR cases.
Another important consideration is data sharing. The authors state that the sequencing data will be uploaded to the ENA repository only after the manuscript is published and will be available for only three years. Ideally, sequence data should be made publicly available without time restrictions to promote transparency and reproducibility.
The authors should expand on the following limitations in the discussion:
-The small sample size and its implications for the study’s conclusions.
-The limited sensitivity of tNGS for certain resistance mutations, especially for bedaquiline.
-The potential underestimation of RR cases due to the selection of pre-identified resistant samples and the inability to detect rpoB_I491F with the method used.
The manuscript is a valuable contribution as a descriptive report of the implementation of a commercial tNGS test (Deeplex MycTB) in a high-burden TB setting. However, given its focus on public health implementation rather than novel scientific

insights or robust statistical analysis, the manuscript might be better suited for a journal with a public health orientation.

Reviewer #3

(Remarks to the Author)
I co-reviewed this manuscript with one of the reviewers who provided the listed reports. This is part of the Nature Communications initiative to facilitate training in peer review and to provide appropriate recognition for Early Career Researchers who co-review manuscripts.

Version 1:

Reviewer comments:

Reviewer #1

(Remarks to the Author)
The authors have expanded the sample size, enhanced the presentation of genotypic resistance data and improved the Discussion section. The issues raised in the prior review have been sufficiently addressed in the revisions and responses.

Reviewer #2

(Remarks to the Author)
File attached

Reviewer #3

(Remarks to the Author)
I co-reviewed this manuscript with one of the reviewers who provided the listed reports. This is part of the Nature Communications initiative to facilitate training in peer review and to provide appropriate recognition for Early Career Researchers who co-review manuscripts.

Open Access This Peer Review File is licensed under a Creative Commons Attribution 4.0 International License, which permits use, sharing, adaptation, distribution and reproduction in any medium or format, as long as you give appropriate credit to the original author(s) and the source, provide a link to the Creative Commons license, and indicate if changes were made.
In cases where reviewers are anonymous, credit should be given to 'Anonymous Referee' and the source.
The images or other third party material in this Peer Review File are included in the article's Creative Commons license, unless indicated otherwise in a credit line to the material. If material is not included in the article's Creative Commons license and your intended use is not permitted by statutory regulation or exceeds the permitted use, you will need to obtain permission directly from the copyright holder.
To view a copy of this license, visit https://creativecommons.org/licenses/by/4.0/

Response to Reviewer Comments

We thank the reviewers for their valuable and constructive feedback. Below are point-by-point responses to each comment. We have revised the manuscript accordingly.

Reviewer Comment	Response	Location in Manuscript
It would be helpful to know the average turnaround time for the tNGS results and CAC recommendations.	Thank you very much for the comment. However, due to technical problems in the implementation phase, we are not able to calculate realistic estimates with respect to time to result or time to clinical response. We have added this as a limitation.	Discussion, Page 19, Line 442-445
At what point in treatment were the regimen changes based on tNGS made?	As above, we have added this as a limitation. It varied; majority were before treatment completion (during continuation phase for those on RHZE).	Discussion, Page 20, Line 447-448.
Please explain how indeterminate RIF phenotypic DST results were defined and whether an indeterminate result was associated with the presence of the I491F mutation.	We thank the reviewer for this comment. Indeed, phenotypic DSTs were only performed via MGIT. Using this system there is not an indeterminate value for RIF phenotypic DST but rather an invalid result. The critical concentrations for all of the drugs tested were aligned with those currently recommended by the WHO. We have updated the text to reflect this and specifically note the critical concentration used for rifampicin and bedaquiline in the methods. We also changed the classification in supplemental table S1 to "Invalid= contamination or under-inoculation errors, after the test has been reaped only once"	Laboratory Methods, Page 7, Line 168-172<br>Table S1, page 38
If phenotypic bedaquiline DST or MIC testing was performed on some or all of the isolates, those results would be welcome.	Bedaquiline susceptibility testing was performed via both tNGS and liquid pDST in 84 unique participant strains. Liquid pDST interpreted the majority of strains identified with Rv0678 mutations by tNGS as susceptible. A description of the results by the two methods has been added to the results section, accompanied by a brief rationale for the discordance in the discussion. Of note, the majority of BDQ	Results, Page 13, Line 300-308<br><br>Discussion, Page 18, Line 399-404.

	resistant strains have two Rv0678 mutations M146T, and N98D, for which MIC testing has been done in a previous paper showing a MIC increase in line with what has been described for other clinically relevant Rv0678 mutations	
A perspective on the perceived sustainability of the program in Eswatini would be welcome in the discussion	A discussion of sustainability and emerging evidence on the cost effectiveness of tNGS in high burden settings has been added to the discussion.	Discussion, Page 20, Line 448-454.
Publication of CAC job aids and SOPs in supplemental material would be valuable.	The job aids and SOPs have been included in the supplemental materials to complement the figure currently in the main text.	Supplemental Materials, page 37
Introduction – Paragraph 2, last sentence: reference needed for this statement	This reference has been added.	Introduction, Page 4, Line 111
Introduction- Paragraph 3, first sentence: the claim that the I491F mutation is not detected by phenotypic assays should be more nuanced	We agree and have specifically included an explanation in the introduction section for the difficulty to detect I491F mediated RIF resistance by liquid phenotypic drug susceptibility testing. Our data clearly confirms this; in strains where both MGIT pDST for rifampicin and tNGS were performed, MGIT identified resistance in only 2 of 90 strains with the I491F strain present and one of those strains also had another rpoB mutation (H445D).	Introduction, Page 4-5, Line 114-118.
Materials and methods Line 2: "endorsed" or "recommended" by WHO may be more appropriate, as WHO does not "approve" products	This has been changed to recommended.	Methods, Page 6, Line 142
Programmatic treatment, first sentence: CAC should be spelled out with	This has been spelled out.	Methods, Page 8, Line 194

first use		
Results<br>Clinical integration of tNGS, first line: what is meant by "sensitization"?	We have modified the wording to improve clarity.	Results, Page 11, Line 245-246.
Results – Initial tNGS results, second paragraph, line 8: "20% (1 of 5) as drug-resistant…" should read "20% (1 of 5) as MDR…"?	The data has been updated, but the text is in line with these recommendations.	Results, Page 12, Line 268-274
Penultimate paragraph: please explain why report forms were not received from 7 of 37 patients and why a recommendation was provided by the CAC for one patient. Of the 15 patients in which a change was recommended, how many actually had a change made in their regimen?	This reflects the programmatic conditions in which this intervention was set. Some clinicians did not complete and submit the forms, opting instead to continue with the regimens they already prescribed which they deemed effective In the broader dataset the CAC provided recommendations for 96% of patients for whom there was complete information provided. The CAC recommended a new regimen in 26 instances. In most cases (22/26), clinicians acted on the recommendations. For 3/26 the clinician had already made the same change recommended by the CAC and one patient died before the recommendations were received. This has been updated with additional details in the results.	Results section Page 15, Line 325-334.
Discussion – Paragraph 3, line 5: suggest to replace "ineffective due to" with "expected to be ineffective due to" or "prescribed despite" or something similar to emphasize that not all mutations confer absolute resistance and effectiveness was not measured	The sentence in the discussion has been reworded to "We found that 32% to 50% of patients would have received an incorrect regimen based on the results from Xpert Ultra and XDR, LPA or MGIT individually or a composite of the standard test results."	Discussion, Page 17, Line 378-379
Please explain why the RIF susceptibility results in Fig 2A and	To more clearly deliver the message being conveyed in Figure 2A we have now restricted to 99 participants (strains) with	Figure 2A-C, Legends, Page

Fig 3A-C do not align in terms of the proportions that are susceptible, resistant, indeterminate and missing.	valid rifampicin resistance results by Xpert Ultra, LPA, pDST, and tNGS. The discordance in resistance detection is clearly evident and reflects the prevalence of the I491F mutation. Figure 3A-C has been replaced with table 2 that clearly describes the number of participants that received each individual test as part of their routine care.	35-36, Line 692-717<br><br>Table 2, Page 26, Line 584-584.
Fig 1B legend: please describe the meaning of the blue vs. orange boxes	This has been added. The color reflects algorithmic responses to resistant vs. susceptible results.	Figure 1B Legend, Page 31, Line 659-661.
Fig 3 legend: what is the "Bottom Row" that is referred to? Did the authors intend to refer to Left and Right columns instead of Top and Bottom rows, respectively? Yellow shading in the Left columns is not defined in the legend. The light blue and dark blue shading described in the legend is not apparent in the figure	This figure has been replaced with table 2 to reflect the larger cohort of participants. We have addressed the ambiguity that this comment highlighted.	Table 2, Page 26, Line 585.
Reviewer 2: We thank Reviewer #2 for their critical insights. We have revised the title, abstract, and discussion to better align with the scope and limitations of the study. Detailed responses follow		
While the study addresses an important topic, the title does not fully reflect the scope or findings, as the small sample size limits a comprehensive evaluation of the clinical impact of tNGS.	We have substantially expanded the resistance results reported. The tNGS results now represent 53% of patients treated in Eswatini over a 3-year period. We have updated the title to reflect the scope of resistance data, highlighting bedaquiline resistance, and the adoption of tNGS as a programmatic tool. We have reduced the emphasis on clinical impact due to the lack of availability on time to tNGS result.	Title, page 1<br>Results section, Page 12, Line 257-274
The abstract does not clearly state the primary objective of the study, and the conclusion regarding	We have reworded the abstract to describe our primary focus as reporting tNGS results as compared to results generated by existing drug susceptibility test platforms in Eswatini and secondarily to describe the	Abstract, Page 2-3, Line 56-73

the alarming situation of bedaquiline resistance is not supported by the data	introduction and use of tNGS as a clinical tool in Eswatini. To our knowledge this remains the first example in a high-burden, low resource setting of tNGS being integrated into routine clinical care. We have also expanded our cohort to 234 samples and samples with bedaquiline resistance were 87, translating to 37%, which is quite high.	
The authors cannot extrapolate these results to claim that bedaquiline resistance is an alarming issue in the country, as the study lacks a population-based design.	While we acknowledge that this is not a population-based study, we now present results from a substantially larger cohort. We have removed the word 'alarming' but do believe that this is a concerning finding, particularly given the detection of the I491F strain throughout the region. and its association with bedaquiline resistance. The latest national DRS (unpublished yet) reported that 60% of I491F strains harbored resistance conferring BDQ mutations. Global TB report in 2019, box 3.4, page 57 also highlighted the prevalence of I491F mutation in Eswatini.	Abstract, Page 2-3, Line 56-73.<br><br>Discussion, Page 16, Line 359-363.
The study analyzed 37 samples, representing only 27% of the patients (119) recruited and approximately 15% of the MDR/RR cases (~240) estimated in Eswatini according to WHO's 2021-2022 report. This small sample size limits the generalizability of the findings. Consequently, conclusions about mutation prevalence should be drawn cautiously.	As above we have substantially increased the size of the sample; however, we have recognized that this is not a population-based study as a limitation in the discussion and have tempered our conclusions regarding mutation prevalence.	Results section, Page 12, Line 257-266.<br><br>Discussion Page 19, Line 457-470.
Statements such as "The extraordinarily high rate of potential bedaquiline and clofazimine resistance is alarming" should be revised. These conclusions, based on a	This has been revised to "is worrisome". We have also substantially increased the size of the sample to 234 strains, thus providing a much more robust dataset supporting our conclusions. And we are confident, that the statement is valid considering the high prevalence of Bedaquiline resistance is 55% in RR strains.	Abstract, page 2, Line 56-66, 69-73.<br><br>Discussion, Page 16, Line 359-363.

limited dataset, carry significant public health implications and risk overstating the findings. The manuscript primarily presents descriptive results as percentages based on a small sampling, without any statistical analysis or indicators of implementation success.		
The mutation analysis is not sufficiently detailed. It is unclear which mutations are missed by each diagnostic test (it can be assumed that the 17 cases with rpoB_I491F are missed by Xpert Ultra, but an additional case is missing) and how rifampicin resistance mutations, such as rpoB_I491F, co-occur with mutations conferring resistance to other drugs like bedaquiline.	We have included supplementary documentation reflecting the results of Xpert Ultra, Xpert XDR, LPA, and MGIT pDST when available as compared to tNGS results. Figure 2C (upset plot) demonstrates how frequently the mutations associated with BDQ resistance occur with the I491F mutation. This is complemented by text describing the proportion of strains with rv0678 mutations.	Supplementary tables page 38.<br><br>Fig 2C, page 35, Line 692-717.
The authors should provide additional context for including Rv0678_N98D as a resistance-associated mutation, given that the WHO mutation catalogue classifies it as Uncertain (WHO-UCN-TB-2023.7).<br>.	We included the reference for the paper in which we did MIC testing of strains from Eswatini with Rv0678_N98D. They indeed show the same level of MIC increase as strains with M146T mutation. 10.1186/s13073-020-00793-8. We also included a section in the discussion on this.	Results Page 13, Line 300-308.<br><br>Discussion, Page 17-18, Line 386-394, Line 399-404.
The manuscript needs a more comprehensive discussion of the limitations of tNGS, particularly regarding its sensitivity for detecting mutations	We have expanded on this point as an additional limitation. However, this does not really apply to the Eswatini setting as there are two dominant Rv0678 mutations which were classified resistant. In addition, additional grading criteria are now published additionally enhancing the	Discussion, Page 17, Line 389-394.

associated with bedaquiline resistance, which is estimated at only ~30% by the WHO catalogue	sensitivity of sequencing based BDQ resistance prediction	
The selection of samples pre-identified as resistant to isoniazid (INH) and/or rifampicin (RIF) introduces bias, as rpoB_I491F—a critical mutation in Eswatini—is not detectable by the methods used. This could lead to an underestimation of RR cases.	We agree that this is a potential concern and we have added it as a limitation. The placement of tNGS at that location in the diagnostic algorithm did take into consideration cost and sequencing capacity; however, it was also based on data from the prior population-based drug resistance survey, which indicated that 94% of strains with the I491F mutation conferring resistance to RR also had isoniazid resistance.	Discussion, Page 20, Line 460-466.
Another important consideration is data sharing. The authors state that the sequencing data will be uploaded to the ENA repository only after the manuscript is published and will be available for only three years. Ideally, sequence data should be made publicly available without time restrictions to promote transparency and reproducibility.	The sequencing data for the 234 strains has been updated to the ENA repository.	Data Sharing, Page 22, Line 503-510.
The authors should expand on the following limitations in the discussion:<br>-The small sample size and its implications for the study's conclusions.<br>-The limited sensitivity of tNGS for certain resistance mutations, especially for bedaquiline.<br>-The potential underestimation of RR	The sample size for the study has been substantially increased but we have tempered our conclusions with respect to the impact on patient outcomes. The limitation regarding the sensitivity of tNGS for bedaquiline resistance detection and the potential under estimation of the prevalence of the I491F mutation have been added. This, however, does not apply to the Eswatini study setting because of the limited mutation diversity.	Results, page 12, Line 257-266<br><br>Discussion, Page 17, Line 389-393,<br>Page 20, Line 457-470.

cases due to the selection of pre-identified resistant samples and the inability to detect rpoB_I491F with the method used		
The manuscript is a valuable contribution as a descriptive report of the implementation of a commercial tNGS test (Deeplex MycTB) in a high-burden TB setting. However, given its focus on public health implementation rather than novel scientific insights or robust statistical analysis, the manuscript might be better suited for a journal with a public health orientation.	We thank the reviewer for the consideration; however, we are confident, that the paper combines clinical, public health, and research aspects in a unique manor, this fitting very well in scope of Nature communications in our view.	
We thank Reviewer #3 for co-reviewing and providing thoughtful evaluation. We acknowledge their contribution to the peer review process.		

Response to Reviewers

Nature Communications — Manuscript Revision

Reviewer Comment 1

Please clarify in the Methods whether all patients included in the study were new TB cases. If not, indicate the proportion of new versus previously treated patients, and specifically report the proportion of drug resistance among retreatment cases.

Response:

Thank you for this important comment. Among the 442 DR-TB cases in the full programmatic cohort (2021–2024), prior treatment status was inconsistently captured in the programmatic database, limiting our ability to report this accurately for the cohort. This is a recognized challenge in programmatic DR-TB data from high-burden settings, where treatment history documentation is frequently incomplete.

Within the tNGS sub-cohort (n=234), prior treatment data retrospectively captured for 147 patients (63%). Of these, 100/147 (68%) had documented prior TB treatment which was limited to first-line therapy (RHZE), 97/100 cases (97%),). This is consistent with the epidemiology of DR-TB in Eswatini, where most resistance is transmitted.

We acknowledge that the incompleteness of programmatic treatment history data is a limitation of this analysis and have noted this explicitly in the limitations section. Given the high proportion of missing data and the risk of introducing systematic bias by reporting a figure based on only 63% of the cohort, we have elected not to report a retreatment proportion; however, we have included this data for the cohort of 59 patients with detailed clinical information available.

Line 477-479:

Further, as is common in programmatic DR-TB cohorts from high-burden settings, prior treatment history was incompletely captured in the surveillance database, limiting our ability to distinguish new from retreatment cases across the full cohort.

Reviewer Comment 2

Regarding the observed increase in bedaquiline resistance, do the authors have information on prior or current bedaquiline exposure? Were these patients treatment-naive, or had they received bedaquiline previously? In other words, were the BDQ-resistance-associated mutations acquired with or without documented BDQ exposure?

Response:

Thank you very much for the question. Indeed, the main driver of BDQ resistance in the cohort is the transmission of MDR strains with *rpoB* I491F mutation with an additional BDQ resistance mutation. These strains were already detected in the DR survey in 2008/2009 and their emergence

is most likely not linked to BDQ exposure (Beckert et al. 2020 10.1186/s13073-020-00793-8.). With the introduction of BDQ treatment, these strains have a likely selective advantage beyond that conferred by the I491F mutation alone and are increasing in the MDR strain population in Eswatini. To make this point clearer, we added the following text to the results section lines 291 to 295.

Overall, 87 isolates were classified as BDQ resistant, out of which 69 had a Rv0678 M146T and 16 had Rv0678 N98D mutation, all of which found in MDR strains with *rpoB* I491F mutation (65 with I491F mutation only, four with an I491F|S450L double mutation) (Supplemental Table 2).

We added the following passage in the discussion line 374-379.

Among the 48 participants with these mutations and had a documented history of prior TB treatment, only one had prior exposure to bedaquiline, suggesting transmission of resistant strains rather than acquired resistance. This is further underlined by the fact, that 85 out of the 87 BDQ resistant strains had either and a Rv0678 M146T (n=69) or a Rv0678 N98D (n=16) mutation, respectively, all of which are found in an MDR strain with I491F mutation, thus linked with MDR strains with *rpoB* I491F mutations, which have already been described previously.

Reviewer Comment 3

The Results section should focus on samples for which results are available across all resistance diagnostic methods. Other values are descriptive but not directly comparable. The current presentation includes numerous partial comparisons, making the results difficult to follow and interpret.

Response:

We appreciate the reviewer's concern about clarity in presenting comparative results. We agree that the 99 samples with complete data across all three methods (Xpert MTB/RIF Ultra, LPA, and tNGS) provide a robust direct comparison. However, we respectfully suggest retaining the presentation of the larger dataset for the following reasons: First, incomplete diagnostic data reflect the realities of programmatic implementation in resource-limited settings, where not all samples undergo every available test due to specimen availability, test failures, or clinical decision-making algorithms. Second, excluding patients with incomplete diagnostic workups would create a highly selected sub-cohort that does not represent the operational context in which tNGS is actually deployed. Third, many key programmatic findings — including detection of drug resistance missed by routine methods and identification of treatment-altering mutations — occur precisely in cases where routine diagnostics are incomplete or discordant, making these cases essential to understanding tNGS's incremental value. Excluding the majority of our cohort from the main results would substantially diminish the impact and generalizability of our findings for national TB programs considering tNGS implementation. We have noted incomplete data as a limitation in the discussion and have not focused on diagnostic accuracy across the routine tests that were performed. Detailed concordance data is available between each routine test and the tNGS results in Supplementary Tables 5A-F. We believe this approach balances analytical rigor with operational relevance.

Reviewer Comment 4

The association between the rpoB I491F mutation and bedaquiline resistance-associated mutations is not a novel finding. It has already been reported in previous studies from Eswatini and Mozambique, including publications by some of the authors, and is appropriately cited in the manuscript. These findings should therefore not be presented or interpreted as new or original.

Response:

We thank the reviewer for this important point and agree that the association between rpoB I491F and bedaquiline resistance has been previously reported, including in our own prior work. However, we respectfully suggest that our current findings represent a significant extension of these earlier observations. While the association was initially identified in smaller samples, our study demonstrates that this co-occurrence is not sporadic but rather represents the predominant pattern of drug resistance at a national programmatic level in Eswatini. The scale and consistency of this finding across our cohort establishes that the rpoB I491F/bedaquiline-resistant strain has become the dominant circulating drug-resistant lineage nationally, which has critical implications for treatment algorithms and surveillance strategies.

We have revised the manuscript to appropriately acknowledge prior work while emphasizing the progressive nature of the current findings. Discussion line 365-371:

This association has previously been described in studies from Eswatini and neighboring countries, including earlier work from our group. However, our findings extend these observations by demonstrating the programmatic scale and clinical relevance of this resistance pattern during routine implementation of sequencing within the national TB program. Within our cohort, 55% of RR-TB strains carried mutations associated with resistance to both bedaquiline and clofazimine, indicating that this co-occurrence represents a substantial proportion of circulating drug-resistant strains detected through routine care.

Reviewer Comment 5

Table 2 suggests a bias toward INH-resistant cases in routine diagnostic testing, which may explain why most strains harbor the rpoB I491F mutation rather than the more common rpoB S450L mutation, as previously described for Eswatini (PMID: 33239092). Could the authors provide the proportions of drug-susceptible, INH-resistant, and RR/MDR cases identified by routine tests for the full cohort (n = 442) during the study period?

Response:

Thank you for raising this point. The diagnostic algorithm under which tNGS was performed indicates tNGS for any INH resistant sample and any RIF resistant sample detected e.g by Xpert. We could not base our inclusion criterion on RR cases only, as all strains with I491F mutation not detected by Xpert or line probe would have been missed. So, by design our study should reflect an unbiased view of the MDR strain population circulating in Eswatini. However, we agree that we

did not perform a drug resistance survey, and our cohort only includes the samples sent to the NTRL. This limitation is described in detail in the discussion (lines 480 – 487).

Lastly, not all people diagnosed with TB underwent tNGS. The population chosen for tNGS by the diagnostic algorithm may not be indicative of how widespread this problem is (which can only be known through comprehensive active surveillance or formal drug resistance surveys). The diagnostic algorithm that was used to indicate the need for sequencing was dependent on the presence of isoniazid or rifampicin resistance detection. As such it is possible that we are underestimating the prevalence of Mtb strains with the *rpoB* I491F mutation, or that we are overestimating the prevalence if strains detected by Xpert were less likely to be sent to the NTRL.

Reviewer Comment 6

Some comparison with pre-implementation data would be valuable to contextualize the impact of introducing tNGS and should be addressed in the Discussion.

Response:

This is an important point. Treatment success has been increasing, now at 86% compared to 77.1% in 2019 before implementation. The decline in 2022 was likely due to COVID-19 effects on patients enrolled in 2020 and followed during the pandemic. Cure rates declined in 2024, but treatment success remained high due to a rise in 'Completed' outcomes secondary to culture reagent stock-outs. Deaths have been declining during this period.

DR-TB Outcomes

	Year	Treatment Success (cured+ completed)	Cured	Completed	Died	LTFU	Failed
Pre-implementation	2019	77.1%	74.7%	2.5%	15.7%	2.2%	4.7%
	2020	81.1%	78.0%	3.0%	11.1%	2.7%	5.1%
Post-Implementation	2021	80.4%	76.8%	3.6%	11.1%	5.2%	3.2%
	2022	75.2%	69.9%	5.2%	12.4%	6.5%	5.9%
	2023	84.2%	62.4%	21.8%	7.9%	4.2%	3.6%
	2024	86.3%	18.3%*	68.0%*	9.2%	3.9%	0.7%

** Low cure rate in 2024 due to culture reagent stock-outs; rise in 'Completed' outcomes reflects difficulty assigning 'Cured' outcome.*

To clarify this, we have added the above table to the supplement and the following text on lines 352 – 355:

This high treatment success rate has been mirrored by programmatic DR-TB outcomes overall. Treatment success for all DR-TB patients nationally increased from 77.1% in 2019 to 86.3% in 2024, with declines in deaths and treatment failure over the same period (Supplemental Table 6).

We have added the following text on line 385-386:

Beyond our cohort, program data has also demonstrated continuous improvement in successful outcomes, potentially benefitting from the individualized therapy facilitated by tNGS.

Reviewer Comment 7

There is a discrepancy between the Abstract and the Methods regarding the inclusion criteria. Please revise to ensure consistency.

Response:

We have carefully reviewed both sections and revised the Methods to match the Abstract section. We made a minor revision regarding treatment failure as an indication for tNGS; however, we feel that the cohort selection is currently placed more appropriately in the data analysis section of the methods.

The following modification was made: "or suspected treatment failure as requested by treating physician". Abstract, Line 58-59 and Methods-Program Population, Line -154-155.

Reviewer Comment 8

Values reported in Supplementary Table 1 do not fully match those described in the text (e.g., RR detected by Xpert Ultra). Please review all values for consistency throughout the manuscript.

Response:

All results have been reviewed and are now consistent. We have updated the Xpert Ultra results to match the text and corrected several Xpert Ultra results which were misclassified in the original document as resistant. There was discordant between laboratory information system documents and the clinical record, which has now been resolved.

Reviewer Comment 9

The only directly comparable results between methods are those from the 99 strains with complete data across all diagnostic tools. Based on these data:

- What proportion of these 99 strains harbored the rpoB I491F mutation?

- Were all strains missed by routine tests explained by the presence of rpoB I491F, or were other factors involved?

- It would be helpful to explicitly identify rpoB I491F in Figure 2A, as the same number of strains is represented for each method, allowing clearer visualization of the source of discordance.

- References should not be included in this paragraph, as it reports the own results.

Response:

Thank you for noting the need for clarification. Among the 99 strains with complete data across all diagnostic methods, 46 (46%) harbored the rpoB I491F mutation. All strains missed by Xpert

were attributable to the I491F mutation apart from one strain with both I491F and S450L mutations. MGIT and LPA classified the same strain with I491F and S450L mutations as susceptible, in addition to two strains with H445L mutations, otherwise all missed strains were attributable to the I491F mutation. We have revised Figure 2A to explicitly label rpoB I491F-containing strains, allowing direct visualization of this as the primary source of diagnostic discordance.

Reviewer Comment 10

Please ensure consistent definitions of drug resistance by tNGS. According to the Methods, resistance classification relies on the extended Deeplex/WHO catalogue. Mutations not included in the catalogue should not be classified as resistant.

Response:

We state in the methods that “If variants were uncharacterized, polymorphisms were further interpreted using a curated mutation catalogue employed at the Research Center Borstel (v2022-11-22) as described before(13,14)“. This was required because v3 of the deeplex catalogue did not include the most up to date WHO catalogue. This was described in Line 191-193 and was again clarified in the results section on line 232-233:

Eswatini aligned result interpretation with the WHO catalogue of mutations launched in 2021 to define tNGS resistance classifications, which was further updated in 2023(4) and supplemented interpretation with the Research Center Borstel catalogue for rare mutations incompletely addressed in the WHO catalogue.

Reviewer Comment 11

Accordingly, the statement that 22 strains were BDQ-resistant by tNGS but susceptible by pDST is inconsistent with the later clarification that only 20 strains harbor catalogue-listed BDQ-R mutations. By definition, this corresponds to 20 BDQ-resistant strains by tNGS. The authors may additionally note that two strains harbor mutations associated with increased MICs not confirmed by pDST, but consistency in terminology is essential.

Response:

The terminology was consistent with the approach outlined in the methods. The M146T was initially classified as an uncharacterized mutation in the deeplex interpretation v3 catalogue, which we used at that time. The v2 WHO catalogue and later in the v4 deeplex catalogue defined this mutation as resistant. The N98D mutation was not uncharacterized in the deeplex v3 and v2 WHO catalogue; therefore, we reverted to the curated mutation catalogue employed at the Research Center Borstel. We have performed MIC testing in previous published work on strains with the N98D mutation, which had elevated MICs to BDQ. This approach aligns with that outlined in the methods.

Reviewer Comment 12

Can the authors propose any interpretation for the reclassification of MDR/RR cases by routine testing as drug-susceptible by tNGS?

Response:

There were only three strains that were classified as resistant to rifampicin by MGIT, LPA or Xpert but susceptible by tNGS. It is possible this was due to an uncharacterized mutation or a false resistance result by Xpert, LPA or MGIT. Discordance at this rate is common within a programmatic context.

Reviewer Comment 13

The statement that the association between bedaquiline resistance and the epidemic rpoB I491F strain represents a 'major finding' of this study is not supported. This association has already been reported in Eswatini and Mozambique, including prior publications by the same group (PMIDs: 33239092, 37956677), and is correctly cited. It should therefore not be framed as an emerging or novel observation.

Response:

Thank you for the suggestion, we have removed this sentence from the results.

Reviewer Comment 14

Line 321-322: 'of regimens' is repeated; please revise.

Response:

Thank you — we have addressed this typo.

Reviewer Comment 15

The paragraph is confusing, as a few lines above it is stated that CAC recommended treatment changes for 26 patients. The following rephrasing is suggested:

Based on tNGS results, 31/59 patients (53%) had their regimens changed. Of these, 25 changes were made following CAC recommendations, while clinicians independently modified treatment in six cases, suggesting increasing confidence among treating clinicians."

Response:

Thank you for the suggestion — we have modified the language accordingly. Results section, Line 337-339.

Based on tNGS results, 31/59 patients (53%) had their regimens changed. Of these, 25 changes were made following CAC recommendations, while clinicians independently modified treatment in six cases, suggesting increasing capacity and confidence among the treating clinicians.

Reviewer Comment 16

Did the authors observe differences in treatment outcomes between patients whose regimens were changed and those whose regimens were not?

Response:

23/28 (82.1%) of patients who did not change regimens based on tNGS results and (29/31) 93.5% of patients who did change had successful treatment outcomes. Given the observational nature of the cohort and the small sample size we did not emphasize this difference in the manuscript. In the overall cohort, the treatment success rate was 88%, 8% died and 3% were lost to follow-up.

Reviewer Comment 17

How do these outcomes compare with historical data prior to tNGS implementation?

These outcomes compare favorably with the overall programmatic outcomes. Programmatically, treatment success has been increasing, now at 86% compared to 77.1% in 2019 before implementation. The decline in 2022 was likely due to COVID-19 effects on patients enrolled in 2020 and followed during the pandemic. Cure rates declined in 2024, but treatment success remained high due to a rise in 'Completed' outcomes secondary to culture reagent stock-outs. Deaths have been declining over the years. The improvement in programmatic outcomes may reflect the increasing use of tNGS results.

DR-TB Outcomes

	Year	Treatment Success (cured+completed)	Cured	Completed	Died	LTFU	Failed
Before implementation	2019	77.1%	74.7%	2.5%	15.7%	2.2%	4.7%
	2020	81.1%	78.0%	3.0%	11.1%	2.7%	5.1%
During Implementation	2021	80.4%	76.8%	3.6%	11.1%	5.2%	3.2%
	2022	75.2%	69.9%	5.2%	12.4%	6.5%	5.9%
	2023	84.2%	62.4%	21.8%	7.9%	4.2%	3.6%
	2024	86.3%	18.3%*	68.0%*	9.2%	3.9%	0.7%

** Low cure rate in 2024 due to culture reagent stock-outs; rise in 'Completed' outcomes reflects difficulty assigning 'Cured' outcome.*

To clarify this, we have added the following text on lines 348 – 351:

This high treatment success rate has been mirrored by programmatic DR-TB outcomes overall. Treatment success for for all DR-TB patients nationally increased from 77.1% in 2019 to 86.3% in 2024, with declines in deaths and treatment failure over the same period (Supplemental Table 6).

The table has been added to the supplement, and we have added the following line to the discussion on line 383-384:

Program data has also demonstrated continuous improvement in successful outcomes, potentially benefitting from the individualized therapy facilitated by tNGS.

Reviewer Comment 18

Is there evidence that the number of drug-resistant cases has been reduced following tNGS implementation, or is it too early to assess programmatic impact?

Response:

It is too early to assess whether tNGS implementation has reduced the number of drug-resistant TB cases in Eswatini. The study period (2021-2024) represents the initial implementation phase, and the time lag between improved case detection/treatment and measurable reductions in population-level drug-resistant TB incidence generally requires a longer timeframe to observe.

ROUND 2 REVIEWER 2 ATTACHMENT:

This manuscript by Vambe et al. is a substantially revised version of the previously submitted work and should be considered a new manuscript rather than a revision. The authors clearly state that their objective is to describe the development and implementation of a national tNGS program for drug-resistance diagnosis in tuberculosis, as well as the operational experience gained during this process.

While the work is carefully conducted and provides a detailed programmatic description, the study remains largely descriptive, reporting mainly proportions of drug-resistant strains before and after the introduction of tNGS. The manuscript does not include formal statistical analyses, counterfactual comparisons, or pre–post implementation analyses that would allow assessment of the incremental impact of tNGS. As such, the results offer limited conceptual or methodological novelty and are primarily of operational or programmatic interest.

General comments

1. Please clarify in the Methods whether all patients included in the study were new TB cases. If not, indicate the proportion of new versus previously treated patients, and specifically report the proportion of drug resistance among retreatment cases.

2. Regarding the observed increase in bedaquiline resistance, do the authors have information on prior or current bedaquiline exposure? Were these patients treatment-naïve, or had they received bedaquiline previously? In other words, were the BDQ-resistance–associated mutations acquired with or without documented BDQ exposure?

3. The Results section should focus on samples for which results are available across all resistance diagnostic methods. Other values are descriptive but not directly comparable. The current presentation includes numerous partial comparisons, making the results difficult to follow and interpret.

4. The association between the rpoB I491F mutation and bedaquiline resistance–associated mutations is not a novel finding. It has already been reported in previous studies from Eswatini and Mozambique, including publications by some of the authors, and is appropriately cited in the manuscript. These findings should therefore not be presented or interpreted as new or original.

5. Table 2 suggests a bias toward INH-resistant cases in routine diagnostic testing, which may explain why most strains harbor the rpoB I491F mutation rather than the more common rpoB S450L mutation, as previously described for Eswatini (PMID: 33239092). Could the authors provide the proportions of drug-susceptible, INH-resistant, and RR/MDR cases identified by routine tests for the full cohort (n = 442) during the study period?

6. Some comparison with pre-implementation data would be valuable to contextualize the impact of introducing tNGS and should be addressed in the Discussion.

Methods

- There is a discrepancy between the Abstract and the Methods regarding the inclusion criteria. Please revise to ensure consistency.

Results

Lines 268–274

- Values reported in Supplementary Table 1 do not fully match those described in the text (e.g., RR detected by Xpert Ultra). Please review all values for consistency throughout the manuscript.

- The only directly comparable results between methods are those from the 99 strains with complete data across all diagnostic tools. Based on these data:

  - What proportion of these 99 strains harbored the rpoB I491F mutation?
  - Were all strains missed by routine tests explained by the presence of rpoB I491F, or were other factors involved?
  - It would be helpful to explicitly identify rpoB I491F in Figure 2A, as the same number of strains is represented for each method, allowing clearer visualization of the source of discordance.

- References should not be included in this paragraph, as it reports the authors' own results.

Lines 302–308

- Please ensure consistent definitions of drug resistance by tNGS. According to the Methods, resistance classification relies on the extended Deeplex/WHO catalogue. Mutations not included in the catalogue should not be classified as resistant.

- Accordingly, the statement that 22 strains were BDQ-resistant by tNGS but susceptible by pDST is inconsistent with the later clarification that only 20 strains harbor catalogue-listed BDQ-R mutations. By definition, this corresponds to 20 BDQ-resistant strains by tNGS. The authors may additionally note that two strains harbor mutations associated with increased MICs not confirmed by pDST, but consistency in terminology is essential.

- Can the authors propose any interpretation for the reclassification of MDR/RR cases by routine testing as drug-susceptible by tNGS?

Lines 310–318

The statement that the association between bedaquiline resistance and the epidemic rpoB I491F strain represents a "major finding" of this study is not supported. This association has already been reported in Eswatini and Mozambique, including prior publications by the same group (PMIDs: 33239092, 37956677), and is correctly cited. It should therefore not be framed as an emerging or novel observation.

Line 321–322: “of regimens” is repeated; please revise.

Lines 330–332

The paragraph is confusing, as a few lines above it is stated that CAC recommended treatment changes for 26 patients. I suggest the following rephrasing:

> “Based on tNGS results, 31/59 patients (53%) had their regimens changed. Of these, 25 changes were made following CAC recommendations, while clinicians independently modified treatment in six cases, suggesting increasing confidence among treating clinicians.”

Lines 336–343

- Did the authors observe differences in treatment outcomes between patients whose regimens were changed and those whose regimens were not?

- How do these outcomes compare with historical data prior to tNGS implementation?

- Is there evidence that the number of drug-resistant cases has been reduced following tNGS implementation, or is it too early to assess programmatic impact?
