# Supplementary material for: Targeted next-generation sequencing implementation in Eswatini identifies rifampicin and bedaquiline resistance undetected by routine diagnostic testing
Source: Nat Commun. 2026 Jun 11;17:8227. doi: 10.1038/s41467-026-73551-w (PMC13462916; doi:10.1038/s41467-026-73551-w)
Supplement: Supplementary file 3 [file 41467_2026_73551_MOESM3_ESM.pdf]

Description of Additional Supplementary Files

File Name: Supplementary Data 1

Description: Sample-level European Nucleotide Archive (ENA) accession codes and per-drug resistance calls for the 234 sequenced patient samples. Provided as an Excel (.xlsx) file. Columns: SampleID, PatientID, ENA_ACCESSION_NUMBER, Year, Sequencing_result_acceptability, Composite_target_coverage_breadth (%), Average_coverage_depth (x), Species best match, and per-drug resistance calls (RIF, INH, PZA, EMB, SM, FQ, KAN, AMI, CAP, ETH, LIN, BDQ, CFZ; 0 = susceptible, 1 = resistant, "" = no result), Resistance_Category.
