# Supplementary material for: Targeted next-generation sequencing implementation in Eswatini identifies rifampicin and bedaquiline resistance undetected by routine diagnostic testing
Source: Nat Commun. 2026 Jun 11;17:8227. doi: 10.1038/s41467-026-73551-w (PMC13462916; doi:10.1038/s41467-026-73551-w)
Supplement: Supplementary file 4 [file 41467_2026_73551_MOESM5_ESM.pdf]

nature portfolio

Corresponding author(s): Stefan Niemann

Last updated by author(s): Apr 23, 2026

Reporting Summary

Nature Portfolio wishes to improve the reproducibility of the work that we publish. This form provides structure for consistency and transparency in reporting. For further information on Nature Portfolio policies, see our Editorial Policies and the Editorial Policy Checklist.

Statistics

For all statistical analyses, confirm that the following items are present in the figure legend, table legend, main text, or Methods section.

n/a	Confirmed	
☐	☒	The exact sample size (*n*) for each experimental group/condition, given as a discrete number and unit of measurement
☒	☐	A statement on whether measurements were taken from distinct samples or whether the same sample was measured repeatedly
☒	☐	The statistical test(s) used AND whether they are one- or two-sided<br>*Only common tests should be described solely by name; describe more complex techniques in the Methods section.*
☐	☒	A description of all covariates tested
☒	☐	A description of any assumptions or corrections, such as tests of normality and adjustment for multiple comparisons
☒	☐	A full description of the statistical parameters including central tendency (e.g. means) or other basic estimates (e.g. regression coefficient) AND variation (e.g. standard deviation) or associated estimates of uncertainty (e.g. confidence intervals)
☒	☐	For null hypothesis testing, the test statistic (e.g. *F*, *t*, *r*) with confidence intervals, effect sizes, degrees of freedom and *P* value noted<br>*Give P values as exact values whenever suitable.*
☒	☐	For Bayesian analysis, information on the choice of priors and Markov chain Monte Carlo settings
☒	☐	For hierarchical and complex designs, identification of the appropriate level for tests and full reporting of outcomes
☒	☐	Estimates of effect sizes (e.g. Cohen's *d*, Pearson's *r*), indicating how they were calculated

Our web collection on statistics for biologists contains articles on many of the points above.

Software and code

Policy information about availability of computer code

Data collection	Clinical data for the study was collected manually from the clinical records. The specimens for sequencing were analyzed on an illumina iseq platform as described in the manuscript.Sequence analysis and mutation detection were performed using the bioinformatics pipeline on the Deeplex® Myc-TB web application (version: Deeplex Myc-TB V3_0_1 - Extended catalogue). If variants were uncharacterized, polymorphisms were further interpreted using a curated mutation catalogue employed at the Research Center Borstel (v2022-11-22).
Data analysis	The clinical and sequencing data were analyzed descriptively, as was appropriate for a clinical programmatic pilot project.Visualizations of the data were created with R version 4.5.1.

For manuscripts utilizing custom algorithms or software that are central to the research but not yet described in published literature, software must be made available to editors and reviewers. We strongly encourage code deposition in a community repository (e.g. GitHub). See the Nature Portfolio guidelines for submitting code & software for further information.

Data

Policy information about availability of data

All manuscripts must include a data availability statement. This statement should provide the following information, where applicable:

- Accession codes, unique identifiers, or web links for publicly available datasets
- A description of any restrictions on data availability
- For clinical datasets or third party data, please ensure that the statement adheres to our policy

The targeted next-generation sequencing data generated in this study have been deposited in the European Nucleotide Archive (ENA) database under the accession

codes listed in Supplementary Table S7. The clinical and epidemiological data are available under restricted access due to patient data privacy laws. Access to individual participant data can be obtained by submitting a methodologically sound protocol for individual patient meta-analysis to Dr. Debrah Vambe (Debrah.Vambe@bcm.edu). Requests will be reviewed by the Eswatini NTCP and access granted contingent on the execution of appropriate data access agreements

Research involving human participants, their data, or biological material

Policy information about studies with human participants or human data. See also policy information about sex, gender (identity/presentation), and sexual orientation and race, ethnicity and racism.

Reporting on sex and gender	We have described sex appropriately.
Reporting on race, ethnicity, or other socially relevant groupings	All patients were of African and Emaswati heritage.
Population characteristics	We have provided tables on what we considered the relevant clinical co-variates and outcomes for the study population.
Recruitment	Participants were recruited programmatically through identification of drug resistance to isoniazid/rifampicin, or if they were suspected of treatment failure. Verbal informed consent was then obtained for participation in the programmatic pilot. A potential self-selection/referral bias exists, as the algorithm depended on the presence of isoniazid or rifampicin resistance detection; therefore, this cohort may under- or overestimate the true prevalence of the rpoB I491F mutation in the broader population.
Ethics oversight	The protocol for the programmatic implementation of this pilot study was reviewed by the Eswatini Health and Human Research Review Board and the Baylor College of Medicine IRB.

Note that full information on the approval of the study protocol must also be provided in the manuscript.

Field-specific reporting

Please select the one below that is the best fit for your research. If you are not sure, read the appropriate sections before making your selection.

☒ Life sciences ☐ Behavioural & social sciences ☐ Ecological, evolutionary & environmental sciences

For a reference copy of the document with all sections, see nature.com/documents/nr-reporting-summary-flat.pdf

Life sciences study design

All studies must disclose on these points even when the disclosure is negative.

Sample size	This was a programmatic report describing the clinical impact of an established diagnostic tool. No statistical method was used to predetermine sample size. The sample size (n=234 isolates, with n=59 having complete clinical data) was determined by the programmatic availability of patient samples meeting the inclusion criteria between June 2021 and December 2024 at the National Tuberculosis Reference Laboratory and DR-TB sites. This sample size was deemed sufficient to describe the programmatic implementation and diagnostic yield of tNGS within this specific national cohort and worth sharing with the larger medical community. Many other high-burden TB settings are now also considering introducing targeted sequencing and we feel that this example will be illustrative.
Data exclusions	There are no data exclusions.
Replication	The Deeplex assay is a clinically approved and validated test. There was not an indication for performing replicates.
Randomization	This was not a randomized study design.
Blinding	There was no blinding.

Reporting for specific materials, systems and methods

We require information from authors about some types of materials, experimental systems and methods used in many studies. Here, indicate whether each material, system or method listed is relevant to your study. If you are not sure if a list item applies to your research, read the appropriate section before selecting a response.

Materials & experimental systems

n/a	Involved in the study	
☒	☐	Antibodies
☒	☐	Eukaryotic cell lines
☒	☐	Palaeontology and archaeology
☒	☐	Animals and other organisms
☐	☒	Clinical data
☒	☐	Dual use research of concern
☒	☐	Plants

Methods

n/a	Involved in the study	
☒	☐	ChIP-seq
☒	☐	Flow cytometry
☒	☐	MRI-based neuroimaging

Clinical data

Policy information about clinical studies

All manuscripts should comply with the ICMJE guidelines for publication of clinical research and a completed CONSORT checklist must be included with all submissions.

Clinical trial registration	This was not a clinical trial and therefore was not registered as such. The project used standard drugs and regimens certified for treatment of MDR TB. The interpretation of sequencing results was based on a WHO endorsed technology using the WHO resistance catalogue to guide treatment.
Study protocol	The project protocol will be submitted with the manuscript.
Data collection	Data was collected through monitoring and evaluation mechanisms established by the National Tuberculosis Control Program and the Eswatini Health Laboratory Services.
Outcomes	Outcomes for the patients treated using targeted sequencing results were generated programatically, in-line with World Health Organization outcome reporting guidelines.-

Plants

Seed stocks	*Report on the source of all seed stocks or other plant material used. If applicable, state the seed stock centre and catalogue number. If plant specimens were collected from the field, describe the collection location, date and sampling procedures.*
Novel plant genotypes	*Describe the methods by which all novel plant genotypes were produced. This includes those generated by transgenic approaches, gene editing, chemical/radiation-based mutagenesis and hybridization. For transgenic lines, describe the transformation method, the number of independent lines analyzed and the generation upon which experiments were performed. For gene-edited lines, describe the editor used, the endogenous sequence targeted for editing, the targeting guide RNA sequence (if applicable) and how the editor was applied.*
Authentication	*Describe any authentication procedures for each seed stock used or novel genotype generated. Describe any experiments used to assess the effect of a mutation and, where applicable, how potential secondary effects (e.g. second site T-DNA insertions, mosiacism, off-target gene editing) were examined.*
